# Disturbed RNA editing in MORF3-deficient *Arabidopsis* mitochondria leads to impaired assembly of complex I

**DOI:** 10.1093/plphys/kiaf471

**Published:** 2025-09-30

**Authors:** Matthias Döring, Hans-Peter Braun, Nils Rugen

**Affiliations:** Institute of Plant Genetics, Leibniz University Hannover, Herrenhäuser Str. 2, Hannover 30419, Germany; Institute of Plant Genetics, Leibniz University Hannover, Herrenhäuser Str. 2, Hannover 30419, Germany; Institute of Plant Genetics, Leibniz University Hannover, Herrenhäuser Str. 2, Hannover 30419, Germany

## Abstract

Transcripts in plant mitochondria and chloroplasts undergo editing prior to translation, with approximately 500 specific sites edited in mitochondria and about 30 in plastids of the model plant Arabidopsis (*Arabidopsis thaliana*). Although the full role of this mechanism is not yet understood, it is presumed to compensate for unfavorable mutations accumulated over evolutionary periods. It is also conceivable that RNA editing serves a regulatory function, as proteins can be translated from partially unedited transcripts. In this study, we characterize proteins derived from such mitochondrial transcripts. To enrich these proteins, we use an *Arabidopsis* multiple organellar RNA editing factor 3 (MORF3) mutant, which exhibits reduced RNA editing at numerous specific sites. Despite developmental delays, the mutant plants remain fertile. Physiological and biochemical analyses reveal that complex I of the respiratory chain is particularly affected in the mutants. Consistent with these findings, a shotgun proteomic analysis identified proteins originating from partially unedited NADH dehydrogenase subunit 2 (nad2) and nad7 transcripts. Complexome profiling revealed that these proteins integrate into the holo-complex and, to a lesser extent, into the supercomplex formed by complex I and dimeric complex III. Concurrently, known assembly intermediates of complex I are enriched in the mutant. We demonstrate that the disruption of complex I assembly is caused by the absence of editing at specific sites in transcripts encoding the subunits Nad3 and Nad4L. Our results provide deep insights into the molecular consequences of perturbations within the respiratory complex I.

## Teaser

RNA editing at specific mitochondrial transcript sites in *Arabidopsis thaliana* is crucial for complex I assembly and stability, with deficiencies leading to macro- and molecular phenotypes.

## Introduction

While it has been originally presumed that RNA transcripts from DNA merely transmit information encoded by DNA, it is now understood that RNA may be subject to editing. RNA editing describes the modification of transcripts that leads to specific changes in transcript sequences at so-called “editing sites.” Editing of RNAs has been found in many different organisms, such as viruses, bacteria, fungi, animals, and plants. In plants, it was first described for mitochondria (Covello and Gray 1989; Gualberto et al. 1989; Hiesel et al. 1989) and later also for chloroplasts (Hoch et al. 1991). In flowering plants, mitochondrial and chloroplast transcripts are mainly affected by C-to-U RNA editing. Here, cytidines (C) are deaminated into uridines (U). In the model plant *Arabidopsis* (*Arabidopsis thaliana*), 34 C-to-U editing sites were described for chloroplast transcripts and > 500 sites for transcripts of the mitochondria (Chateigner-Boutin and Small 2010; Bentolila et al. 2012, 2013).

The mitochondrial genome of plants primarily encodes subunits of the protein complexes of the mitochondrial respiratory chain as well as proteins of the mitochondrial ribosomes (Unseld et al. 1997; Sloan et al. 2018). Nine subunits of NADH dehydrogenase complex (complex I), which is the first and by far largest protein complex of the respiratory chain, are encoded by the mitochondrial genome in most plants (Braun et al. 2014). The transcripts of these genes are all edited (Giege and Brennicke 1999). In addition, this protein complex consists of about 40 further subunits, which are encoded in the cell nucleus, formed in the cytosol, and transported posttranslationally into the mitochondria (Braun et al. 2014; Ligas et al. 2019; Meyer et al. 2019; Klusch et al. 2021). The transcripts of these genes are not edited. In the mitochondria, the nuclear-encoded and the mitochondrial-encoded subunits of complex I must be assembled to form the holo-complex (Ligas et al. 2019).

The process of RNA editing requires the presence of specificity factors, which are provided in plants in the form of proteins. At the center are nuclear-encoded pentatricopeptide repeat (PPR) proteins (Barkan and Small 2014). In *Arabidopsis*, they are encoded by 481 different nuclear genes, which constitute an especially large gene family (Gutmann et al. 2020). The PPR motif describes a pattern of 35 amino acids that folds into 2 antiparallel α-helices (Small and Peeters 2000). Two amino acid positions, termed “5” and “L,” are essential for the specific 1-to-1 binding to a sequence of ribonucleotides in RNA targets (Knoop 2023). These canonical “P-type” PPRs, however, are not exclusively involved in RNA editing and are also found in other eukaryotic clades (Schmitz-Linneweber and Small 2008; Takenaka et al. 2013; Knoop 2023). Along with the evolution of RNA editing, land plants have developed more complex “PLS-type” PPR proteins. In addition to the canonical 35-amino acid motif, these also contain shorter (S, 31 or 32 amino acids) and longer (L, 35 or 36 amino acids) PPR motifs. These PLS-PPR proteins play an almost exclusive role in C-to-U RNA editing (Small et al. 2020). Many of the PLS-PPR proteins have carboxy-terminal extensions that include highly conserved protein domain modules, termed E1, E2, and DYW (Gutmann et al. 2020). The DYW domain is the actual enzymatically active domain that deaminates cytidine, thereby performing the RNA editing event (Oldenkott et al. 2019; Takenaka et al. 2021).

While in the moss *Physcomitrium patens* all editing factors are complete DYW-type PPR proteins, most angiosperms have RNA-binding proteins that no longer possess their own DYW domain (Knoop 2023). This separation of RNA binding and cytidine deamination necessitates the integration of both functions within so-called editosomes through a diverse set of auxiliary non-PPR proteins of the RNA editing factor interacting proteins (RIP)/multiple organellar RNA editing factor (MORF) (Bentolila et al. 2012; Takenaka et al. 2012), organelle RNA recognition motif (ORRM; Shi et al. 2016a,b), and organelle zinc-finger (OZ; Sun et al. 2015) families. It is important to note that there is not “1” editosome; rather, there seem to be numerous editosome configurations depending on the editing site or the transcript to be edited (Sun et al. 2016). In contrast to knockouts of genes encoding PPR proteins, which affect only single sites, numerous RNA editing sites are affected by knockouts of genes encoding these factors. Particularly strong effects have been observed with the loss of the factors RIP1/MORF8, RIP3/MORF3, and ORRM3 and ORRM4 (Bentolila et al. 2012; Takenaka et al. 2012; Shi et al. 2015, 2016a,b). This strongly indicates that, unlike most PPR proteins, these factors are present in numerous editosome particles (Sun et al. 2016).

The biological role of RNA editing in the mitochondria and chloroplasts of plants is still not fully understood, but it is assumed that the edits compensate for unfavorable mutations that have become established over evolutionary time periods (Small et al. 2020; Knoop 2023). At the same time, it is being discussed whether RNA editing might also have a regulatory function. This is supported by the occurrence of so-called “partial RNA editing,” the observation that editing frequencies at certain RNA positions may vary between tissues, ecotypes, and different growth or stress conditions of plants, as well as their developmental states (Zehrmann et al. 2008; Chateigner-Boutin and Small 2010; Bentolila et al. 2013; Chu and Wei 2020; Zeng et al. 2024). Furthermore, it has been observed that some RNA editing mutants show no or only very mild phenotypes, despite the lack of restoration of amino acids at conserved positions (Okuda et al. 2010; Takenaka et al. 2010; Rüdinger et al. 2011).

Incompletely edited mRNA can be loaded onto mitochondrial ribosomes (Planchard et al. 2018). The translation of incompletely edited mRNAs should give rise to editing-specific proteoforms (referred to as “unedited” or “partially edited proteins” in this manuscript), causing protein heterogeneity at amino acid positions corresponding to partially edited mRNA sites. The term “proteoform” was proposed to refer to all the different molecular forms of a protein product of a single gene that result from genetic variation, alternatively spliced or differently edited RNA transcripts, or posttranslational protein modifications (Smith et al. 2013). Just a few years after the discovery of RNA editing, investigations were conducted to determine whether proteoforms resulting from differential editing accumulate and can be detected. Early studies suggested that subunits of respiratory and mitoribosomal complexes translated from nonedited transcripts do not accumulate due to as-yet-unknown post- or co-translational processes (Bégu et al. 1990; Grohmann et al. 1994; Lu and Hanson 1994; Williams et al. 1998). Other reports have demonstrated that proteins translated from partially edited mRNAs indeed do occur, leading to protein polymorphism, as shown for the small mitoribosomal subunit 12 (Rps12) in petunia and maize (Lu et al. 1996; Phreaner et al. 1996).

Until lately, however, it was unclear whether such proteoforms are incorporated into mitochondrial protein complexes. We recently applied a deep proteomics strategy to screen the *Arabidopsis thaliana* mitochondrial proteome for the occurrence of partially edited proteins (Rugen et al. 2024). The editing state of over 100 C-to-U editing sites was determined at the protein level, revealing that most proteins accumulate in their edited state. However, in some cases, proteins were detected in both their edited and unedited forms. This was the case for open reading frame 25 (ORF25 or subunit b) of the ATP synthase complex and for Rps3 and the large mitoribosomal subunit 5 (Rpl5), both subunits of the mitochondrial ribosome. Interestingly, the partially edited proteins were incorporated into their respective protein complexes (Rugen et al. 2024).

In the present study, we investigate the functional effects of the incorporation of partially edited proteins into the mitochondrial protein complexes in plants. To enrich these proteins, we investigate these effects in the *Arabidopsis rip3-1*/*morf3-1* knockout mutant line, which is known to have reduced editing rates at about 44 positions in a total of 17 different mitochondrial transcripts (Takenaka et al. 2012; Bentolila et al. 2013). For the NADH dehydrogenase subunits 2 and 7 (Nad2 and Nad7), we can detect partially edited proteins that are incorporated into complex I as well as into the I + III_2_ supercomplex. Resulting holo-complexes have lower NADH dehydrogenase activity and are of reduced stability. The assembly pathway of complex I is disrupted at defined steps. The transfer of our data to the known CryoEM structure of *Arabidopsis* complex I (Klusch et al. 2023) enables the identification of specific RNA editing sites that are responsible for the disturbance of complex I assembly.

## Results

### Phenotypic characterization of *morf3-1*

**Figure 1.**
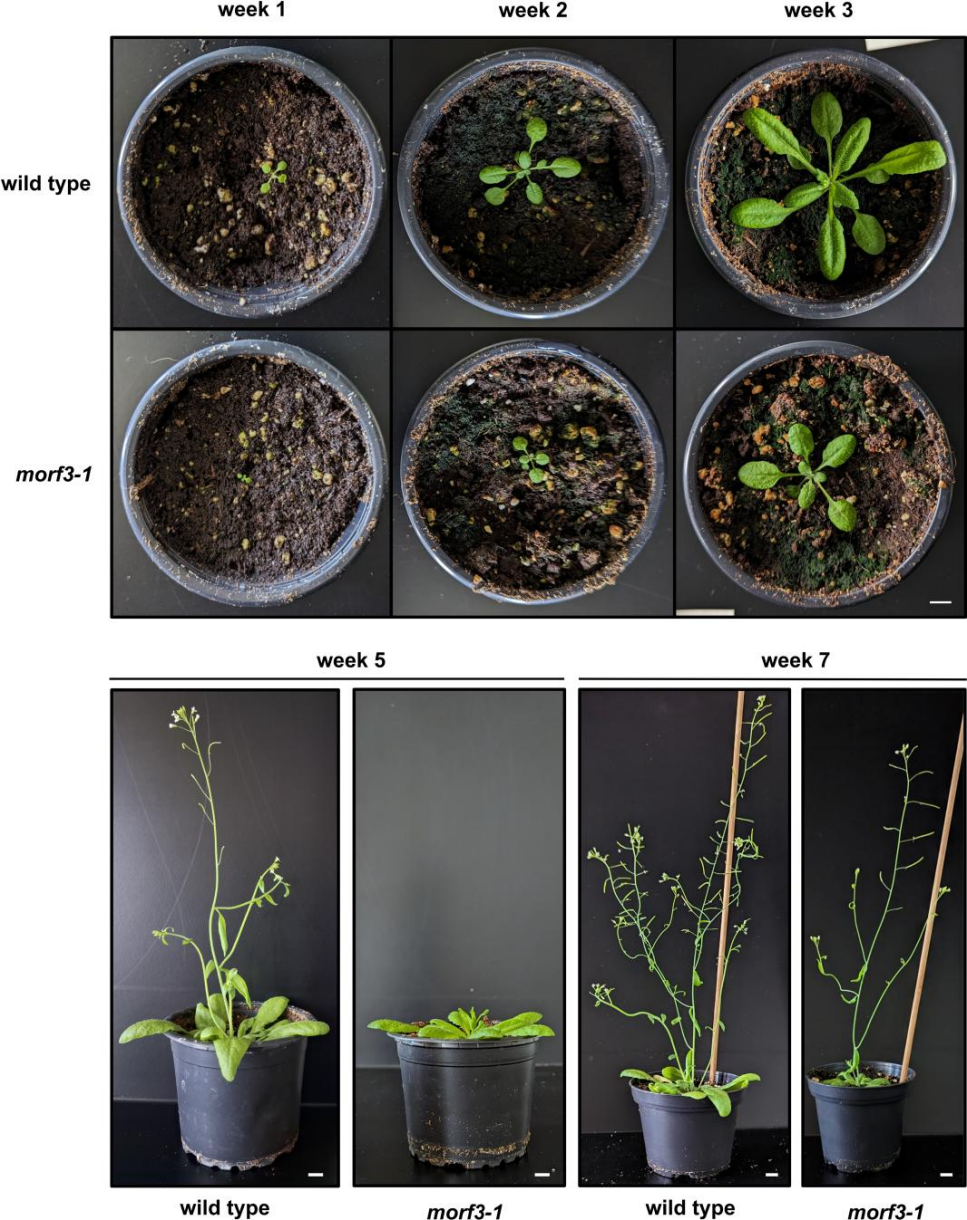
Development of *Arabidopsis thaliana* wild-type and *morf3-1* knockout lines. Plants were grown on soil under long day conditions. Photographs were taken over a period of 7 weeks after germination. White scale bars correspond to 1 cm and are applicable to pictures of the lower or upper panel, respectively.

**Figure 2.**
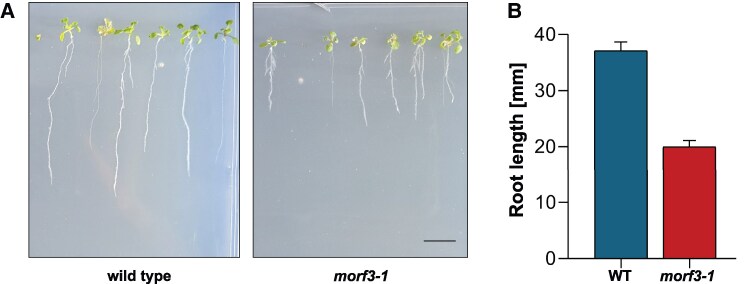
Root growth of *Arabidopsis thaliana* wild-type and *morf3-1* knockout lines. **A)** Root growth upon cultivation of plants on MS-medium under long day conditions. Photographs were taken 10 d after germination. The scale bar indicates a length of 1 cm and is applicable to both images. The root lengths were determined using the ImageJ software package. **B)** Average root lengths of wild-type (WT) and *morf3-1* mutant plants (in mm). Error bars display the standard deviation (WT: *n* = 23; *morf3-1*: *n* = 30).

*Arabidopsis* RIP3/MORF3 (AT3G06790) was first characterized by Takenaka et al. (2012) and Bentolila et al. (2013) and named multiple organellar RNA editing factor 3 (MORF3) or RNA editing factor interacting protein 3 (RIP3) by the authors. We use MORF3 in the following. Knockout lines for the locus AT3G06790 have a phenotype similar to the respective *Arabidopsis* wild-type lines but are slightly delayed in development. We used the *morf3-1* knockout mutant for our study. We started our investigation by characterizing this plant line again phenotypically. Under the applied cultivation conditions, rosette leaves of mutant plants after 3 weeks had approximately the size of the rosette leaves of wild-type plants after 2 weeks (Fig. 1). Inflorescence development started after 4 weeks in wild-type plants but only after 6 weeks in the mutant. The wild-type plants reached fruit maturity after 7 weeks. At this time, the inflorescence of the mutant was still smaller and less branched (Fig. 1). A comparative analysis of root development was carried out by cultivating wild-type and knockout plants on inclined agarose plates. Root growth was substantially delayed in the mutant (Fig. 2). However, the roots in the mutants were more branched.

### Proteomic characterization of *morf3-1*

A transcriptomic study of the *morf3-1* line revealed that the editing rates of mitochondrial transcripts are reduced at about 44 sites (Takenaka et al. 2012). A total of 17 different mitochondrial transcripts are affected, several of which encode subunits of complex I. To investigate the consequences of the reduced editing frequencies at the protein level, a comparative shotgun proteome experiment was carried out using mitochondrial fractions isolated from suspension cell cultures, which were established from the *Arabidopsis* wild-type and *morf3-1* mutant lines (for our experimental setup, see Supplementary Fig. S1). Proteins of mitochondrial fractions from both lines were extracted and digested with trypsin to obtain peptides for subsequent LC-IMS-MS/MS analyses.

Shotgun proteome analyses for both mitochondrial fractions were carried out in biological triplicates. Overall, 6,614 different protein groups were reported (the term “protein group” refers to different types of proteins, each of which is encoded by its own gene. In some cases, however, proteins occur in isoforms and cannot be differentiated on the basis of identified peptides. In these cases, the isoforms are treated as “1 protein group,” even though they are encoded by more than 1 gene). Proteins were quantified using the MaxLFQ algorithm (Cox et al. 2014; Yu et al. 2021) and subsequently further filtered based on their calculated abundance (“MaxLFQ intensity”). Of the 6,614 protein groups, only those for which a MaxLFQ intensity could be calculated in at least 4 out of the total 6 shotgun experiments were included in the comparison between the 2 mitochondrial fractions. Furthermore, a protein group was required to have a MaxLFQ intensity > 0 in all 3 replicates in either the wild type or the *morf3-1* mutant line. This reduced the number of protein groups to 4,471 (Supplementary Table S1).

To estimate the purity of the mitochondrial fractions, all 4,471 protein groups were assigned to subcellular compartments using the SUBAcon algorithm of the SUBA5 database (Hooper et al. 2017). A total of 1,240 protein groups were assigned to mitochondria. Subsequently, the quantities of the identified proteins (calculated in FragPipe based on protein intensity [top-N method]) were summed up for all subcellular compartments (Supplementary Fig. S2). On average, 71.6% of the cumulative protein quantities were assigned to mitochondria, whereas 28.4% were assigned to other subcellular compartments, in particular chloroplasts (11.1%), cytosol (5.5%), peroxisomes (3.4%), and the endoplasmic reticulum (2.3%). We conclude that our mitochondrial fractions have good purity.

To validate our experimental approach, our proteome data sets were first analyzed for the presence of the MORF3 protein. In the mitochondrial fraction from wild-type plants, numerous MORF3 peptides were identified in all replicates. In contrast, MORF3 peptides were completely absent in the mitochondrial fractions of the *morf3-1* mutant line (Supplementary Table S1).

**Figure 3.**
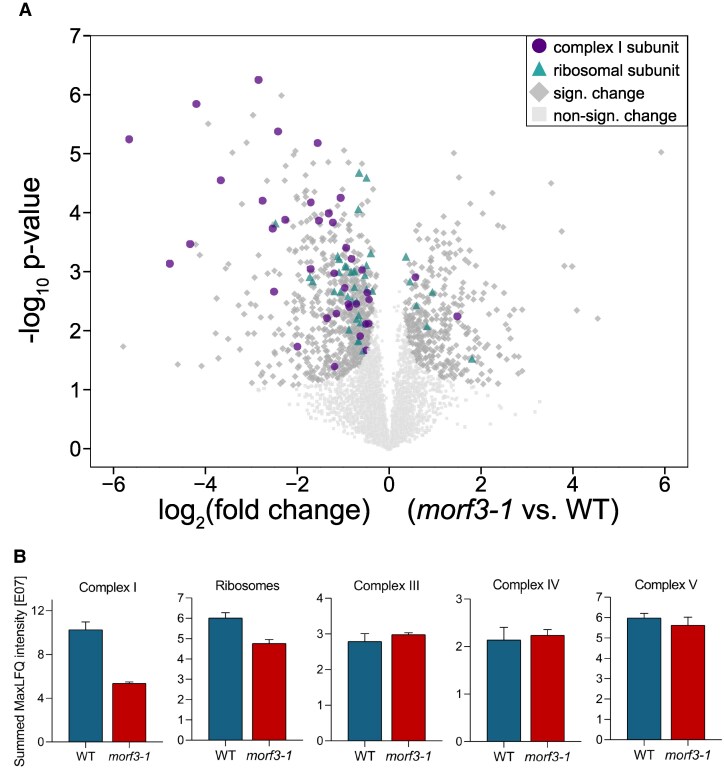
Comparative shotgun proteome analyses of wild-type and *morf3-1* mutant *Arabidopsis* lines. **A)** Volcano plot of differential protein abundance between mitochondrial fractions of wild-type (WT) and *morf3-1* knockout lines (3 independent biological replicates each). The analysis is based on label-free quantification (LFQ). Each dot represents a protein group. The *x*-axis displays the log2 fold difference in abundance between the mitochondrial fractions of the compared lines. Protein groups more abundant in MORF3-deficient plants are shown on the left, while those more abundant in wild-type plants are shown on the right. The *y*-axis displays the -log10 of the *P*-value, reflecting the statistical significance of the observed differences. Proteins with significant differential abundance are highlighted as dark gray diamonds increased in size, while proteins without significant differential abundance are shown as light gray squares. Subunits of complex I with a significant change in abundance are highlighted as purple circles, mitoribosomal proteins as turquoise triangles. **B)** Comparison of summed-up MaxLFQ intensities of subunits of complex I, mitoribosomes, complex III, complex IV, and ATP synthase between wild-type and mutant plants. MaxLFQ values of wild-type plants are displayed in blue, those of the *morf3-1* mutant plants in red. Error bars display standard deviation. Volcano plot generated in Instant Clue (Nolte et al. 2018). Subunits of protein complexes included in our calculation according to Braun et al. (2014) and Waltz et al. (2019).

Differential analysis of the 2 mitochondrial proteomes revealed that MORF3 absence causes dramatic changes (Fig. 3A). Numerous proteins occur in statistically significantly increased or decreased quantities in the mutant. In particular, the subunits of mitochondrial complex I are markedly reduced in the mutant (Fig. 3B). To a lesser extent, subunits of the ribosomes are affected. The quantities of the subunits of the other OXPHOS complexes are largely unchanged. These results are very much in line with the previously reported transcriptome data for the *morf3-1* mutant, which showed that particular transcripts of the mitochondrial complex I genes are less edited in the absence of the MORF3 protein while editing of other OXPHOS components remained mostly unchanged (Takenaka et al. 2012; Supplementary Table S2). We conclude that the *Arabidopsis morf3-1* line has a particular deficiency with respect to complex I.

### Physiological characterization of *morf3-1*

**Figure 4.**
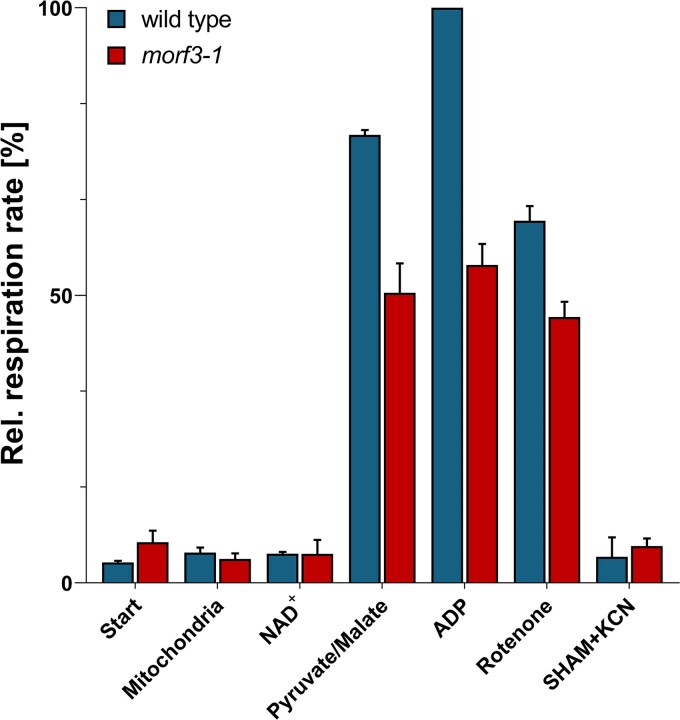
Respiration rates of isolated mitochondria from wild-type and *morf3* mutant *Arabidopsis* lines. Respiration rates were measured by O_2_ consumption/time using a Clark-type oxygen electrode. Freshly isolated mitochondria from 3 independent biological replicates of cell cultures were supplemented with NAD^+^, pyruvate, and malate as substrates and co-substrates for the citric acid cycle, which produces NADH. The NADH provides electrons to the respiratory electron transfer chain, which uses molecular oxygen as a terminal electron acceptor. Coupled to respiratory electron transport, a proton gradient is formed across the inner mitochondrial membrane, which can be used by the ATP synthase complex to form ATP from ADP and inorganic phosphate. Maximal respiration rates (O_2_ consumption per time) are achieved upon provision of ADP. Upon addition of inhibitors, the respiration rates decline. Rotenone inhibits complex I, SHAM blocks the alternative oxidases, and KCN suppresses the cytochrome c oxidase activity. Error bars display the standard error of the mean (SEM) based on 3 technical replicates.

To further investigate the effects of the absence of the MORF3 protein, the respiratory activity of the mitochondrial fractions from the wild-type and *morf3-1* mutant lines was determined using oxygraph measurements (Fig. 4). It was found that the respiration rate of the mitochondrial fraction from the *morf3-1* mutant is reduced by 45% compared to the wild-type line in the presence of substrates for the TCA cycle and ADP. If this measurement was performed in the presence of the complex I inhibitor rotenone, the respiration rates in both lines are reduced, but the difference in the respiration rates is only 17%. We conclude that the overall reduced respiration rate in the mutant is caused in particular by complex I deficiency.

**Figure 5.**
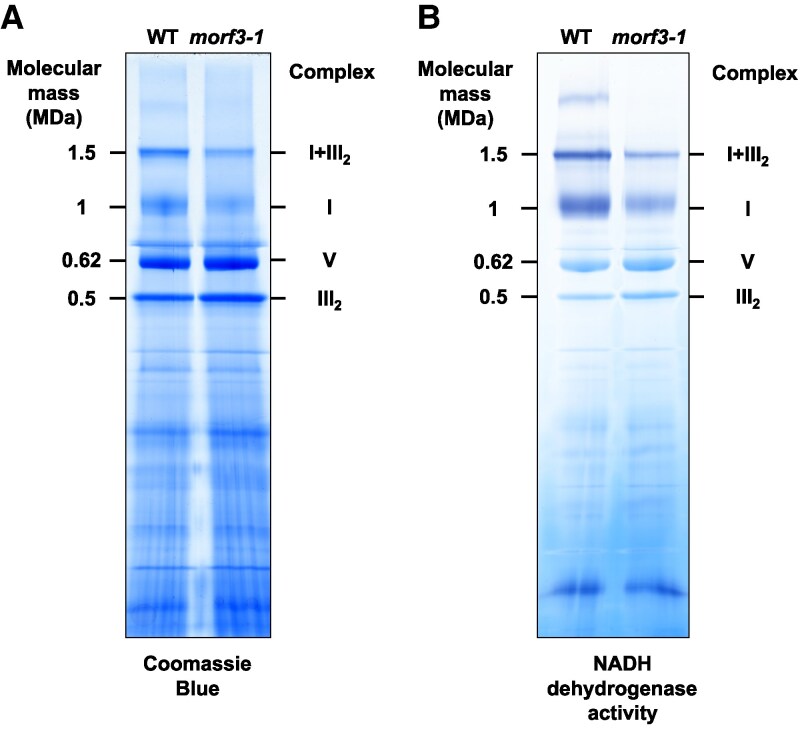
Separation of native protein complexes from mitochondria of wild-type and *morf3-1* mutant *Arabidopsis* lines by blue-native PAGE. Isolated mitochondria from wld type (WT) and *morf3-1* mutant plants were solubilized using 2.5% digitonin. Proteins and protein complexes were separated by blue-native PAGE as described in the “Materials and methods” section. After completion of the gel electrophoresis run, gel lanes were either stained with **A)** Coomassie Brilliant Blue or by an **B)**  *in-gel* NADH dehydrogenase activity assay. A molecular mass scale is displayed to the left of the gels (values in MDa); identities of the protein complexes are given to the right of the gels.

To further explore biochemical differences between the 2 fractions being compared, they were analyzed using 1D blue-native polyacrylamide gel electrophoresis (BN-PAGE) (Fig. 5A). In accordance with the proteomic data, protein complex III_2_ and the ATP synthase complex (complex V) were found to be present in comparable amounts. In contrast, the amount of complex I is reduced in the mutant. Similarly, the amount of a supercomplex consisting of complexes I and III_2_ is reduced in the mutant. An *in-gel* NADH dehydrogenase enzyme assay revealed substantially reduced activity in the *morf3-1* line (Fig. 5B). Thus, our proteomic, physiological, and biochemical results indicate that complex I in particular is affected in the *morf3-1* mutant.

### Detection of “unedited peptides” in *morf3-1* mutant plants

**Figure 6.**
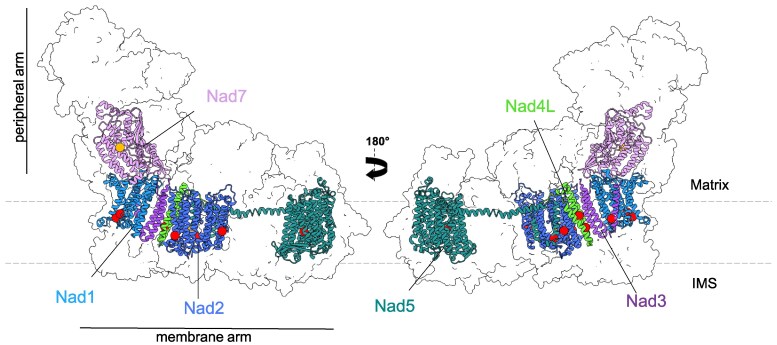
Positions of amino acids affected by reduced RNA editing in *morf3-1* according to Takenaka et al. (2012). Positions of editing sites in Nad1, Nad2, Nad3, Nad4L, Nad5, and Nad7 are indicated by red or orange dots. Only editing sites causing amino acid substitutions are shown (3 of the overall 15 editing positions are “silent”). The 2 sites indicated by orange dots are covered by peptides in our MS dataset. The atomic models of the 6 Nad subunits and the shape of complex I are taken from Klusch et al. (2023), which are available in the Protein Data Bank (PDB) under the ID 8BPX. The boundaries of the inner mitochondrial membrane are indicated by dashed lines. IMS, intermembrane space. Figure generated in ChimeraX (Meng et al. 2023).

With regard to complex I, the transcriptomic analysis of the *morf3-1* mutant showed that mitochondrial transcripts of 6 out of 9 complex I subunits (Nad1, Nad2, Nad3, Nad4L, Nad5, and Nad7) are edited at a reduced rate at a total of 15 sites (Takenaka et al. 2012). Editing at 12 of these sites causes actual amino acid substitutions while 3 positions are “silent.” The positions of the substitution sites can be displayed in the atomic model of *Arabidopsis* complex I (Klusch et al. 2023), and the majority of them are located in a central region of the membrane arm of complex I (Fig. 6).

The question arises whether the 6 complex I transcripts affected in the *morf3-1* mutant are translated and whether proteins from partially edited transcripts are incorporated into complex I. The incorporation of partially edited proteins into protein complexes has indeed recently been demonstrated for the ATP synthase complex and for subunits of the mitoribosomes in *Arabidopsis* wild-type plants (Rugen et al. 2024). To address this question, we reanalyzed our proteomic data of the *morf3-1* mutant line for the presence of peptides covering the amino acid sequences matching the 12 nonsilent editing sites. Since these peptides are derived from proteins translated from either fully or partially edited mRNA, these “editing-specific” peptides can exist in both edited and unedited forms. The sole difference lies in the specific amino acid whose identity is affected by whether the respective RNA has been edited or not.

For 2 different editing events, corresponding peptides were found in the *morf3-1* mitochondrial fractions (Table 1, Supplementary Table S2 and Supplementary Figs. S3 to S6): (i) nad2eU821SFp100 (nomenclature according to Rüdinger et al. 2009: Nad2 subunit, C-to-U editing at position 821, thereby changing the encoded amino acid from serine [S] to phenylalanine [F], editing rate at this site in wild-type *Arabidopsis* plants: 100% according to Bentolila et al. 2013) and (ii) nad7eU739LFp100 (Nad7 subunit, C-to-U editing at position 739, thereby changing the encoded amino acid from leucine [L] to phenylalanine [F]; editing rate at this site in wild-type *Arabidopsis* plants: 100% according to Bentolila et al. 2013) (Table 1). The relatively low coverage of editing sites within the 6 complex I subunits (2 out of 12) can probably be explained by the fact that the Nad subunits are extremely hydrophobic and most peptides are therefore difficult to ionize, which is a prerequisite for their detection by mass spectrometry.

**Table 1.** Identified peptides covering amino acid positions affected by differential editing in morf3-1 mutant plants

Transcript affected	Editing site	aa	Editing freq. WT	Editing freq. *morf3-1*	Edited peptide	Unedited peptide
nad2 (ATMG00285)	nad2eU821SFp100	274	100%	0%	ISI**F**ANILR	ISI**S**ANILR
nad7 (ATMG00510)	nad7eU739LFp100	247	100%	0%	AAPYDVYDQLD**F**DVPVGTR	AAPYDVYDQLD**L**DVPVGTR
rps4 (ATMG00290)	rps4eU299PLp60	100	60%	0%	TSYIPF**L**LNLETR	TSYIPF**P**LNLETR

For each peptide, the identity of the encoding transcript, the editing site, and the amino acid (aa) sequence are provided. The RNA editing frequencies in wild-type and *morf3-1* mutant plants are taken from (Takenaka et al. 2012). Amino acids affected by differential editing are shown in bold. An overview of all RNA editing sites affected by MORF3 deletion is given in Supplementary Table S2.

In *morf3-1*, transcripts edited at the 2 positions were not detectable, meaning that the editing was completely inhibited (Takenaka et al. 2012). In line with these data, only the unedited peptide covering editing site nad2eU821SFp100 was found in the *morf3-1* mutant line. In contrast, both the edited and the unedited peptide were found for the editing site nad7eU739LFp100 in the mutant line. This indicates that editing at this site can still take place in the *morf3-1* mutant line. We assume that the transcriptome analysis did not have sufficient sensitivity to detect the edited transcripts.

### “Partially edited” Nad2 and Nad7 subunits are assembled into the complex I holo-complex

**Figure 7.**
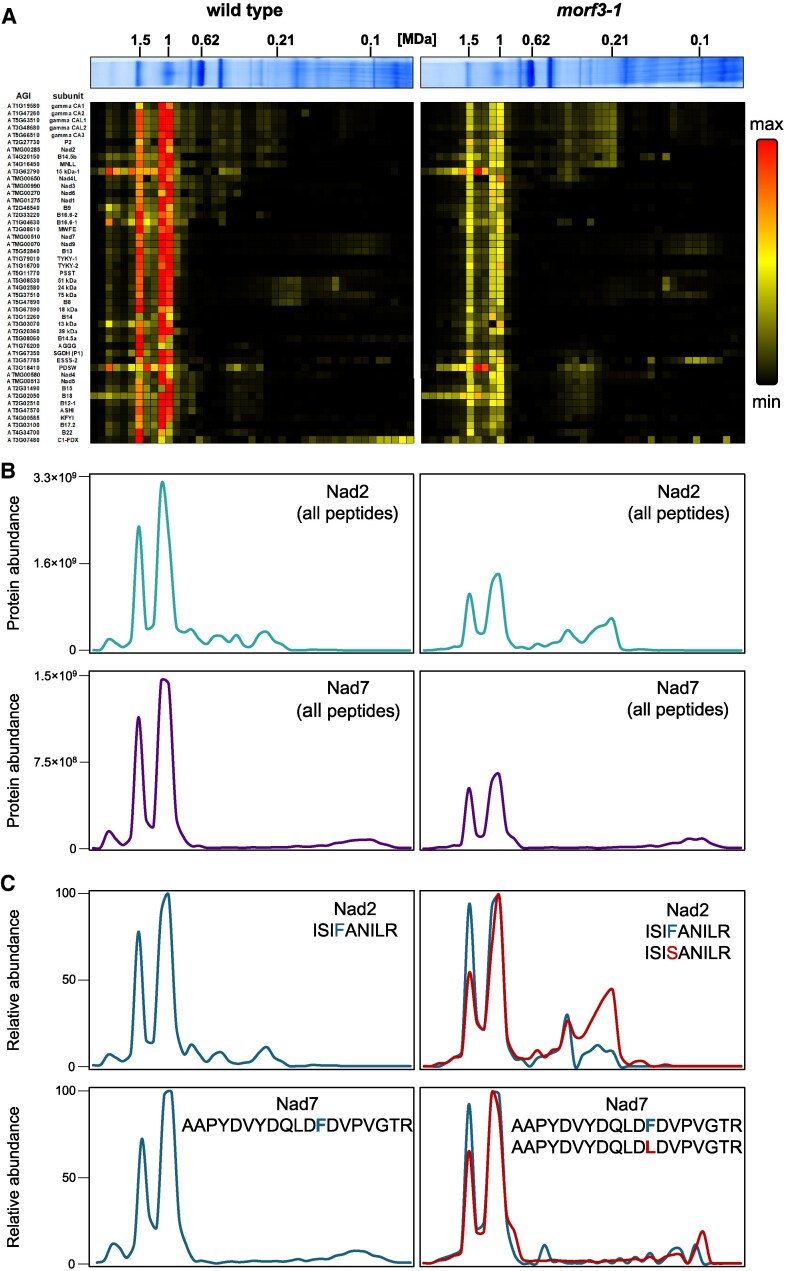
Abundance profiles of complex I subunits along BN gel lanes in wild-type and *morf3-1* mutant *Arabidopsis* lines. Mitochondrial protein fractions from *A. thaliana* wild-type and *morf3-1* mutant liquid cell cultures were separated on a BN gel. The gel lanes shown in Fig. 5A were segmented into 43 fractions and analyzed via mass spectrometry. **A)** Heatmap of proteins of mitochondrial protein complex I from wild-type (left) and *morf3-1* knockout (right) lines. Each column represents a fraction of the BN gel. The rows describe the normalized abundance profiles of individual proteins along the gel stripes. The scale bar on the right indicates protein abundance: red for maximum abundance, yellow for 50% intensity, and black for no detection. Proteins were sorted with respect to their occurrence in the assembly of the complex I according to Ligas et al. (2019). AGI notation and identities of subunits are indicated left of the heatmap. The gel lanes on top of the heatmaps are identical to those shown in Fig. 5A. An accessible version of this complexome heatmap for individuals with color vision deficiency can be found in Supplementary Table S4. **B)** Nad2 and Nad7 abundance profiles based on the summed-up intensities of all Nad2 or Nad7 peptides detected in the wild-type and *morf3-1* knockout lines along the respective gel lanes. Turquoise graph: summed-up intensity profile of all Nad2 peptides. Purple graph: summed-up intensity profile of all Nad7 peptides. The *y*-axis scale applies to both panels. **C)** Normalized abundance profiles of editing-specific Nad2 and Nad7 peptides detected in the wild-type and *morf3-1* knockout lines along the respective gel lanes. The relative abundances for each peptide were normalized to their respective maximum values (“100”). Blue graphs: intensity profiles of edited peptides. Red graphs: intensity profiles of the unedited peptides. The peptides ISIFANILR and ISISANILR can be assigned to the editing site nad2eU821SFp100 of the Nad2 transcript. The peptides AAPYDVYDQLDFDVPVGTR and AAPYDVYDQLDLDVPVGTR can be assigned to the editing site nad7eU739LFp100 of the Nad7 transcript. Nomenclature of RNA editing sites according to Rüdinger et al. (2009).

To test whether partially edited Nad2 and Nad7 proteins enriched in the *morf3-1* mutant are also incorporated into complex I, a complexome profiling experiment was performed (experimental setup in Supplementary Fig. S1): Mitochondrial fractions of the *Arabidopsis* wild-type and the *morf3-1* mutant lines were each separated by BN-PAGE. Subsequently, the gel strips were cut into small pieces from top to bottom, and all pieces (termed “gel fractions”) were analyzed by shotgun proteomics. This results in abundance profiles of mitochondrial proteins along the blue-native gel strips, which can be visualized in the form of heat maps and aligned according to their similarities (Fig. 7A).

In the *Arabidopsis* wild-type line, peptides of Nad2 and Nad7 occur almost exclusively in complex I, as well as in the I + III_2_ supercomplex (Fig. 7, A and B, left panel, respectively). Smaller amounts of peptides of these proteins are present in smaller protein complexes, which likely are assembly intermediates of complex I (Fig. 7B, left panel). In the *morf3-1* mutant, overall amounts of complex I and the I + III_2_ supercomplex are reduced (Fig. 7, A and B, right panel). Peptides of the Nad2 and Nad7 proteins also occur predominantly in the intact complex I and in the I + III_2_ supercomplex. However, the occurrence of Nad2 peptides in assembly intermediates is increased in the *morf3-1* line while no such difference between the wild type and the *morf3-1* mutant line was observed for Nad7 (Fig. 7B, right panel).

If only peptides covering the 2 editing sites are considered, it can be observed that the different Nad2 and Nad7 proteoforms are not incorporated to the same extent in the *morf3-1* mutant (Fig. 7C, upper right panel). For Nad2, it is evident that the unedited proteoform accumulates more substantially in a potential assembly intermediate at approximately 200 kDa compared to the edited proteoform. Both proteoforms peak in the monomeric complex I; however, only the edited proteoforms exhibit a similarly high abundance in the I + III_2_ supercomplex. Nad7 exhibits a similar pattern; however, unlike Nad2, it does not show a marked accumulation in an assembly intermediate (Fig. 7C, lower right panel). We conclude that the Nad2 and Nad7 proteins are incorporated into complex I and the I + III_2_ supercomplex in partially edited form, but that the assembly of complex I is disturbed and consequently assembly intermediates are relatively enriched.

Considering that transcripts of the cytochrome b subunit of complex III (COB), the subunit III of the cytochrome oxidase complex (COX3) and the ORF25 subunit of complex V (also termed subunit b of complex V) are also affected by reduced RNA editing in the *morf3-1* mutant, we examined our complexome profiling data for potential changes in these complexes as well (Supplementary Fig. S7). No differences were observed, further indicating that RNA editing disruptions in the *morf3-1* mutant primarily affect complex I.

### “Unedited” subunits impair the assembly of complex I

The assembly of complex I has already been intensively investigated (Meyer et al. 2011, 2019, 2022; Ligas et al. 2019). Complex I consists of 2 large elongated regions called arms (the peripheral arm and the membrane arm) that are assembled into an L-shaped particle. Both arms are composed of 2 modules, the peripheral arm of the NADH-oxidation (N) module and the quinone reduction (Q) module; the membrane arm, which in its entity is called the proton translocation (P) module, is composed of 2 submodules, 1 proximal to the peripheral arm (P_P_ module) and 1 distal with respect to the peripheral arm (P_D_ module).

**Figure 8.**
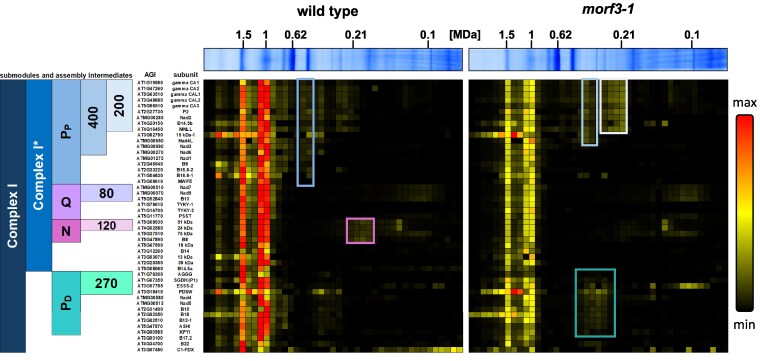
Complex I assembly intermediates in wild-type and *morf3-1* mutant *Arabidopsis* plants as displayed by complexome profiling. Heatmaps as shown in Fig. 7. The gel lanes on top of the heatmaps are identical to those shown in Fig. 5A. Heatmap to the left: abundance profiles of complex I subunits along a blue-native gel stripe of a mitochondrial sample from an *Arabidopsis* wild-type line. Heatmap to the right: abundance profiles of complex I subunits along a blue-native gel stripe of a mitochondrial sample from the *morf3-1* mutant line. For further information on the maps, see legend of Fig. 7. Complex I subunits are sorted according to their presence in complex I assembly intermediates (Ligas et al. 2019). Colored blocks to the left of the heatmaps illustrate assembly intermediates (submodules) of complex I as described before (Ligas et al. 2019); designations and molecular masses (in kDa) of the assembly intermediates are given within blocks. *Arabidopsis* gene identifiers (AGIs) and names of the subunits are indicated in-between the assembly intermediate illustration and the 2 heatmaps. Assembly intermediates of increased abundance in one or the other mitochondrial sample are indicated by colored frames within the heatmaps (the colors of the frames correspond to those in the assembly intermediate [submodule] illustration to the very left of the figure). An accessible version of this complexome heatmap for individuals with color vision deficiency can be found in Supplementary Table S4.

The modules described above are assembled from even smaller units of 80, 85, 120, 200, 270, and 400 kDa (scheme to the left in Fig. 8) and finally built together in a defined sequence of events (Meyer et al. 2011, 2022; Ligas et al. 2019). The subunit composition of the assembly intermediates is largely known (Meyer et al. 2011; Schimmeyer et al. 2016; Ligas et al. 2019; Soufari et al. 2020). An analysis of our complexome profiling data in this regard now shows that 3 defined intermediates are present in the *morf3-1* mutant in increased amounts (Fig. 8, Supplementary Fig. S8): the complete P_D_ module and 2 subcomplexes of the P_P_ module (200 and 400 kDa). Upon closer examination of the supposed 400 kDa intermediate, it appears to contain little Nad3 and no Nad6. In contrast, the complete P_P_ module is of decreased abundance in the *morf3-1* line, as well as the N module. We conclude that the presence of partially edited subunits impairs the assembly of complex I at defined steps, leading to the accumulation of specific assembly intermediates.

### The insertion of partially edited subunits reduces the thermal stability of complex I

**Figure 9.**
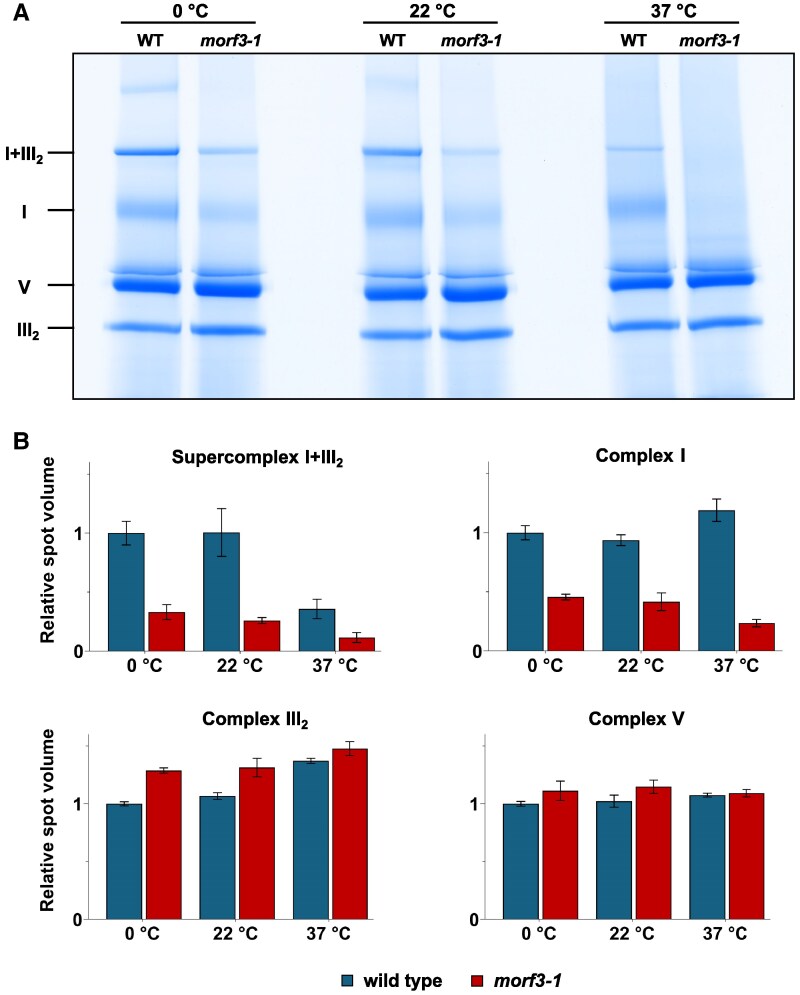
Temperature stability of mitochondrial protein complexes in wild-type and *morf3-1* mutant *Arabidopsis* lines. Freshly isolated mitochondria from both plant lines were incubated at different temperatures (on ice, 22 °C, 37 °C) for 30 min. **A)** Analysis of protein fractions by BN-PAGE and Coomassie staining. Identities of the OXPHOS complexes are given to the right. **B)** Quantification of protein complexes on the gels by Delta2D evaluation. Data are based on BN gels of 3 independent mitochondrial isolations. Protein complex quantity is given as “relative spot volume” (normalized with respect to the quantity of the complexes in the wild-type line after treatment on ice). Error bars display the standard deviation.

Partially edited proteins can be incorporated into holo-complex I. To test the stability of the resulting protein complex, mitochondrial fractions from *Arabidopsis* wild-type and *morf3-1* mutant lines were subjected to heat treatment, and the integrity of the respiratory chain complexes was subsequently checked by 1D BN-PAGE (Fig. 9A). Heat treatments were 22 and 37 °C for 30 min; control samples were kept on ice for the same time period. In both the wild-type and the *morf3-1* mutant, a decrease in I + III_2_ supercomplex abundance was observed after incubation at 37 °C, while only a minor decrease was detected after incubation at 22 °C (Fig. 9B). Caused by the disintegration of the I + III_2_ supercomplex, an increase in the abundance of monomeric complex I is expected. This trend is evident in the wild type after incubation at 37 °C. However, in the *morf3-1* mutant, the level of monomeric complex I decreases after incubation at 37 °C. Simultaneously, the abundance of the dimeric complex III increases in both the wild-type and the *morf3-1* mutant as expected. We conclude that the incorporation of partially edited proteins compromises the thermal stability of complex I while the minor reduction in the editing of the cob (ATMG00220) transcripts (80% compared to 100%; Supplementary Table S2) does not affect the thermal stability of complex III. Similarly, the stability of complex V is hardly affected by the heat treatment in the *morf3-1* mutant line.

## Discussion

### A proteomic investigation of the *morf3-1* mutant

MORF3 is required for efficient RNA editing at 44 sites on a total of 17 different mitochondrial transcripts in *Arabidopsis thaliana* (Takenaka et al. 2012). Several of these transcripts encode subunits of complex I. However, it was not known whether partially edited transcripts are translated into proteins and whether these proteins are used for the formation of complex I and with what consequences. A proteomic analysis of the *morf3-1* mutant was used here to investigate the effects of reduced editing at the protein level. Our results show that partially edited proteins can be inserted into complex I but that this incorporation has numerous negative consequences: (i) the assembly of complex I is disturbed, (ii) the amount of complex I is reduced, (iii) the NADH dehydrogenase activity is additionally decreased, and (iv) the holo-complex has a reduced stability. A protein complex can be seen as a 3-dimensional puzzle. If RNA editing does not take place, the pieces of the protein puzzle may no longer fit together perfectly. This makes it more difficult to assemble the puzzle and leads to a less stable and active product. It can be concluded, at least for complex I of the respiratory chain of *Arabidopsis thaliana*, that fully edited transcripts are a prerequisite for proper complex I function and assembly.

Our proteome dataset contained only peptide sequences matching to 2 of the 12 nonsilent editing sites present in complex I transcripts, which are characterized by a reduced editing rate in the *morf3-1* line (Supplementary Table S3; Takenaka et al. 2012). For both sites, peptides derived from unedited transcripts were predominantly found, in agreement with the corresponding transcriptome data (Takenaka et al. 2012). However, a few peptides derived from the edited forms of the transcripts were also found. We conclude that in the *Arabidopsis morf3-1* line, editing at the 2 sites is severely reduced, but not by 100%. We cannot provide information on the editing rate in the *morf3-1* mutant line at the other 10 sites relevant for complex I. A deep proteomics approach, based on the parallel digestion of proteins of interest with several endoproteases, could provide clarification on the editing status of further complex I subunits and subunits of other complexes affected by MORF3 (Rugen et al. 2024).

It can be assumed that in the *morf3-1 line*, transcripts with editing sites not covered by peptides are also translated and resulting proteins are incorporated into their protein complexes or their assembly intermediates. This primarily appears to affect the assembly and stability of complex I. Deletion of the MORF3 gene probably leads to a multiply altered complex I with overall negative consequences. Nevertheless, complex I is formed and shows activity, albeit to a reduced extent. Mutant plants have a lower respiration rate (Fig. 4); therefore, less energy and consequently grow more slowly, which explains their developmental delay.

However, the consequences of editing events concerning complex I transcripts, which are not covered by corresponding peptides in our proteome study, can be modulated on the basis of the known CryoEM structure of *Arabidopsis* complex I (Klusch et al. 2023).

### The assembly of complex I in the *morf3-1* line is predominantly disturbed at 1 defined step

**Figure 10.**
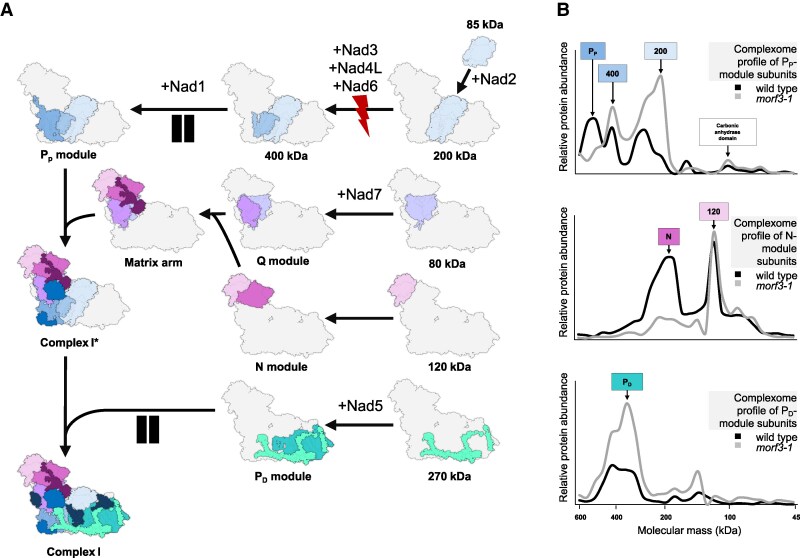
Complex I assembly and its disturbance in the *morf3-1* mutant line. **A)** Complex I assembly is illustrated in accordance with reports in the literature (Ligas et al. 2019). The colors of the assembly intermediates correspond to those given in Fig. 8; the positions of the assembly intermediates within the complex I holo-complex are indicated by their arrangement in relation to the known structure of the *Arabidopsis* complex I (Klusch et al. 2023); the order of steps during complex I assembly is indicated by black arrows. Nad subunits are highlighted at the stages where they are integrated. A step specifically affected in the *morf3-1* mutant line is indicated by a red lightning, and 2 subsequent disturbed or stalled steps are indicated by a pause symbol. **B)** Relative abundance profiles of complex I submodules within the < 600 kDa range of the BN gel stripes upon complexome profiling analysis as given in Figs. 7 and 8 (see Supplementary Fig. S7 for full heatmaps of the < 600 kDa range). The graphs display the summed-up abundance profiles of the subunits assigned to the P_P_ module, the P_D_ module, the Q module, and the N module. Black graphs display abundance profiles in mitochondrial fractions of wild-type plants; gray graphs display those in the *morf3-1* mutant plants. The colored boxes above the graphs include the names of the modules (the colors correspond to those of the complex I assembly intermediates shown in part A of the figure). The complex I assembly pathway in part A of the figure was generated with PyMOL (the PyMOL Molecular Graphics System, version 3.1, Schrödinger, LLC).

The assembly of complex I is disturbed in the *morf3-1* mutant line (Fig. 8). To further evaluate which steps in complex I assembly are affected in the mutant, the complexome profiles of subunits constituting the complex I modules and other known assembly intermediates were integrated (Fig. 10B). The accumulation of assembly intermediates indicates that they are either unable to form successive larger intermediates or that they remain in a “standby mode” until additional, not yet constructed assembly intermediates, are available for integration. A reduced abundance of assembly intermediates may suggest a disturbed formation of these intermediates. Alternatively, it could indicate enhanced degradation of incompletely assembled subunits or a decrease in their synthesis or import as proposed for nonassembled nuclear-encoded subunits of the matrix arm (Lim et al. 2016; Ligas et al. 2019).

**Figure 11.**
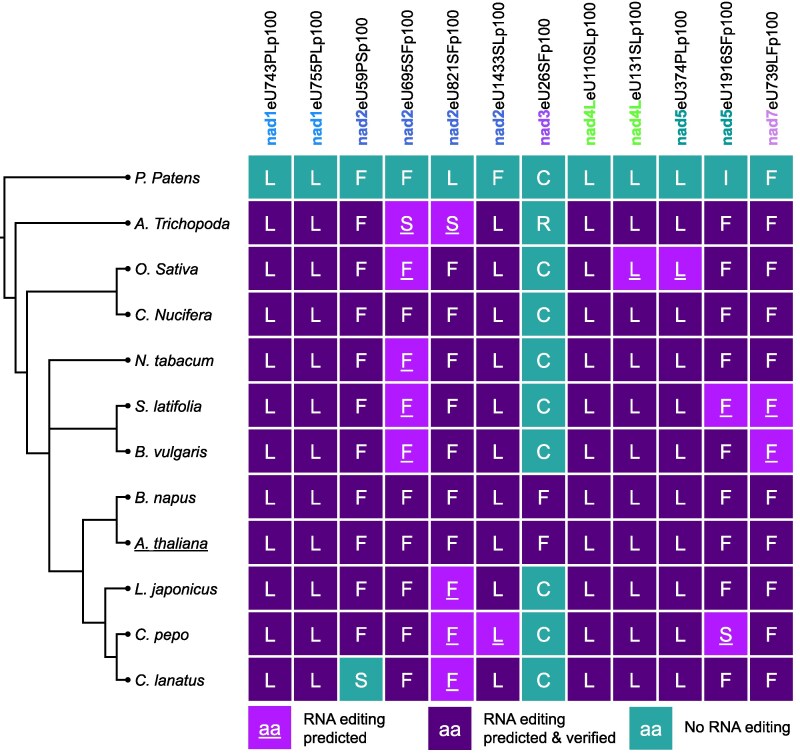
Conservation of MORF3-dependent RNA editing sites on complex I transcripts in various angiosperms. The different plants are arranged in a cladogram based on phylogenetic data from the NCBI taxonomy database (Schoch et al. 2020). Tree branch lengths are not proportional to evolutionary distance. The moss *P. patens* serves as a phylogenetically distant reference. None of the 12 sites presented here is edited in *Physcomitrium*. In contrast, in all angiosperms, RNA editing takes place at almost all of the 12 positions. Column headers refer to the editing positions in *Arabidopsis thaliana* and are in the same order as in Supplementary Table S3. The encoded amino acids of the transcripts are given in the 1-letter code. Experimentally verified editing sites are highlighted in dark purple, predicted editing events in light purple, and nonedited sites in turquoise. RNA editing site predictions as given by the PREPACT portal (Lenz et al. 2018).

The accumulation of the P_D_ module in the *morf3-1* mutant line indicates that it can be fully assembled in this mutant. The module also includes the Nad5 subunit, whose transcripts are edited less efficiently at 2 sites in the *morf3-1* mutant. Since both sites are conserved as most of the other editing sites affected in the *morf3-1* mutant (Fig. 11), they likely play a crucial role in the assembly, stability, or activity of complex I. Editing event nad5eU374PLp100 causes removal of a proline from an alpha helix, which likely is necessary for the correct folding of Nad5 (Maldonado et al. 2022; Supplementary Fig. S9). Editing event nad5eU1916SFp100 causes substitution of a hydrophilic serine with a hydrophobic phenylalanine. This site is located in the C-terminal arm of Nad5, which extends toward the P_P_ module and likely facilitates interaction with Nad2 and hence might be necessary for the assembly of the P_D_ module with the complex I* intermediate (Supplementary Fig. S9). However, both sites are still edited at an editing frequency of 50% to 60%, respectively, in the *morf3-1* mutant (Takenaka et al. 2012; Supplementary Table S3). We hypothesize that the remaining fully edited transcripts are sufficient to synthesize an adequately large population of fully edited and functional Nad5 proteins with small subpopulations of unedited proteins. It has indeed been demonstrated that for most mitochondrially encoded proteins, only the edited proteoforms accumulate in detectable amounts, even when the editing frequency ranges from 60% to 100% (van Wijk et al. 2024). We therefore conclude that the rather modest reduction of editing of Nad5 transcripts is not primarily responsible for triggering the phenotype observed in the *morf3-1* mutant.

Another observation made in the *morf3-1* mutant line was the reduced abundance of the assembled N module of the matrix arm. Since this module lacks mitochondrially encoded Nad subunits, the cause of its decreased abundance must originate elsewhere. During the assembly of the matrix arm, the N module is combined with the Q module (Ligas et al. 2019; Fig. 10A). In *morf3-1*, the conserved editing event nad7eU739LFp100 on Nad7 transcripts is no longer edited to a detectable level (Fig. 11; Supplementary Table S3). Editing at this site causes substitution of a leucine by a phenylalanine and was reported not to be critical for interactions with other subunits but to be situated near key functional domains (Maldonado et al. 2022). Our analysis of the complex I structure also suggests that this editing site does not play a crucial role in the assembly of the matrix arm (Supplementary Fig. S10) which is further supported by the fact that the unedited proteoform can be incorporated into the holo-complex and the supercomplex (Fig. 7). We instead hypothesize that the decreased levels of the N module could result from a feedback mechanism that downregulates the expression and/or import of its subunits. Quantitative protein turnover assessments (Huang et al. 2020) could reveal whether biosynthesis is downregulated or if protein degradation is upregulated, possibly as a protective measure to prevent an accumulation of unassembled matrix arm modules within mitochondria as suggested before for human mitochondria (Lim et al. 2016).

Unlike the P_D_ module, the P_P_ module only shows minimal accumulation. Starting from the 85 kDa intermediate, also known as the carbonic anhydrase (CA) domain, additional subunits are incorporated to form the 200 and 400 kDa intermediates, ultimately resulting in the assembly of the 450 kDa P_D_ module (Ligas et al. 2019; Fig. 10A). Besides several nuclear-encoded subunits, it comprises the 5 mitochondrially encoded subunits Nad1, Nad2, Nad3, Nad4L, and Nad6, with reduced transcript editing observed for all subunits except Nad6 in the *morf3-1* mutant. During the assembly of the 200 kDa intermediate, Nad2 is the first mitochondrially encoded protein to be incorporated, a process also observed in the *morf3-1* mutant (Figs. 7 and 8). Four editing sites on nad2 transcripts fail to reach 100% editing frequency in the *morf3-1* mutant (Supplementary Table S3). However, 2 of these sites maintain an editing frequency of 80%, suggesting, as with Nad5, that the edited proteoforms represent the predominant Nad2 proteoform. The remaining 2 sites both substitute serines with phenylalanines at amino acid positions 20 and 274. The latter site was covered by editing-specific peptides and is situated at the center of Nad2 and in proximity to a functionally important residue (Maldonado et al. 2022; Supplementary Fig. S11A). The other site still exhibits an editing frequency of 20% and is located at an interface with the nucleus-encoded subunit MNLL, also called the 20.9 kDa subunit (At4g16450) (Supplementary Fig. S11B). Since both Nad2 and MNLL are present in the 200 kDa intermediate (Supplementary Fig. S8), it appears that neither unedited site impairs its assembly. However, it is notable that primarily the unedited Nad2 proteoform carrying a serine instead of a phenylalanine at position 274 is present in the 200 kDa intermediate (Fig. 7). This might suggest that the editing of 1 site or a combination of sites not covered by our proteomic approach is necessary for Nad2 incorporation into the 400 kDa intermediate.

**Figure 12.**
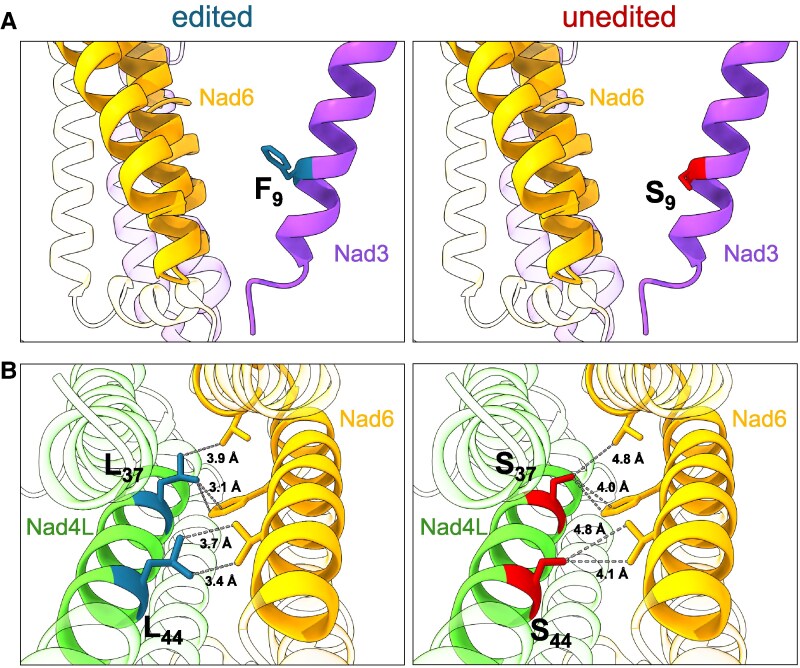
Effects of disturbed Nad3 and Nad4L transcript editing on the structure of complex I in the *morf3-1* mutant line. **A)** Editing at the site nad3eU26SFp100 causes a serine (red, right panel) to be replaced by a phenylalanine (blue, left panel) at amino acid position 9 in the Nad3 subunit (purple). This amino acid position is located at the interphase of Nad3 and Nad6 (yellow). Editing frequency at this site is reduced by 50% in the *morf3-1* mutant line (Takenaka et al. 2012). **B)** Editing at sites nad4LeU110SLp100 and nad4LeU131SLp100 causes 2 adjacent serine residues (red, right panel) to be replaced by leucine residues at amino acid positions 37 and 44 (blue, left panel) in the Nad4L subunit (green). These sites are located at the interface of Nad4L and Nad6 (Maldonado et al. 2022). Distances between the side chains at this interface are given in angstroms. The reported remaining RNA editing frequencies in the *morf3-1* mutant are 0% at nad4LeU110SLp100 and 70% at nad4LeU131SLp100 (Takenaka et al. 2012). This figure displays the protein structures based on the data from Klusch et al. (2023), which are available in the Protein Data Bank (PDB) under the ID 8BPX.

In contrast to the 200 kDa intermediate, a fully assembled 400 kDa intermediate is absent in the *morf3-1* mutant (Fig. 8 and Supplementary Fig. S8). The formation of the 400 kDa intermediate involves the binding of subunits Nad3, Nad4L, Nad6, and the assembly factor GLDH to the 200 kDa intermediate (Fig. 10A; Ligas et al. 2019). In the *morf3-1* complexome, what appears to be the 400 kDa intermediate contains Nad4L and small amounts of Nad3. However, Nad6 could not be detected in this region of the complexome, suggesting that its interaction with the yet incomplete 400 kDa intermediate is compromised. In the *morf3-1* mutant, 3 editing sites in the transcripts encoding Nad3 and Nad4L, which cause substitution of hydrophilic amino acids with hydrophobic ones, are edited at reduced frequency. Notably, all 3 amino acid positions are located at interfaces with Nad6 (Maldonado et al. 2022; Fig. 12; Supplementary Table S3). However, only 1 site, nad4LeU110SLp100, is edited to an undetectable level in the *morf3-1* mutant line (Supplementary Table S3). This causes a decrease in hydrophobicity at the interface as well as a greater distance between the amino acid side chains of both proteins (Fig. 12). We hypothesize that it is primarily serine at position 37 of Nad4L that disrupts the incorporation of Nad6 in MORF3-deficient mitochondria and that this is the main reason for impaired assembly of complex I in the mutant line.

The importance of this particular site is further highlighted in mutants lacking the RNA editing factor slow growth 2 (*SLO2*), which also shows reduced levels of complex I. In this mutant, besides editing event nad4LeU110SLp100, only 2 other nonsilent events are heavily disrupted: nad7eU739LFp100 and mttBeU145PSp100 (Zhu et al. 2012). All 3 events are also disturbed in the *morf3-1* mutant (Supplementary Table S2). Given that editing event nad7eU739LFp100 is most likely not essential for assembly but function (Maldonado et al. 2022; Supplementary Fig. S10; Fig. 8), we suspect that in the *slo2* mutant, it is also primarily the lack of editing at site nad4LeU110SLp100 that leads to the reduced amount of intact complex I (Zhu et al. 2012). The third editing event, mttBeU145PSp100, results in the substitution of an amino acid in the twin arginine translocase C (TatC, also known as OrfX or Mttb) protein, which is thought to be a subunit of the plant mitochondrial TatBC translocase complex (Carrie et al. 2016; Schäfer et al. 2020). However, this protein has not yet been detected by mass spectrometry and is probably present at extremely low amounts (van Wijk et al. 2024). We were unable to find peptides of this protein in either the wild-type or the *morf3-1* line. BN-PAGE and western blot analysis revealed that in the *slo2* mutant, complexes III and IV are also compromised due to the absence of SLO2 (Zhu et al. 2012). As no alterations in the editing of complex III or IV transcripts were observed in the study, it was suggested that the deficiency in complex III and IV could be attributed to a reduced incorporation of specific subunits, resulting from a lack of functional TatC protein. Since complex III remains intact in our *morf3-1* mutant under normal conditions (Fig. 3; Fig. 5) and even after heat treatment (Fig. 9), a similar malfunction of TatC in the *morf3-1* mutant appears unlikely. As the SLO2 study used the harsher detergent dodecyl maltoside (DDM), while this study employed digitonin, it is possible that the instability of complex III in the *morf3-1* mutant might only become apparent after incubation with DDM rather than digitonin. However, this is not the case, as comparative BN-PAGE analysis revealed no observable detergent-dependent differences in the abundance of OXPHOS complexes between mitochondria of *Arabidopsis* wild-type and *morf3-1* mutant lines (Supplementary Fig. S12). A comparative proteomic analysis of the *slo2* and *morf3-1* mutant lines could provide insights into whether both mutants are disrupted in the same complex I assembly step.

Since the formation of the P_P_ module is hindered by the absence of the 400 kDa intermediate, we cannot determine whether partially edited Nad1 proteoforms affect the assembly of complex I. Structural analysis suggests that the folding of Nad1 may be impacted, although neither site is directly involved in the binding of other subunits (Supplementary Fig. S13).

### The effect of the *morf3-1* mutation on other mitochondrial protein complexes

In *morf3-1*, editing rates of transcripts coding for subunits of further protein complexes are reduced, e.g. of some ribosomal transcripts. Our shotgun proteomic data indicate that mitochondria in the *morf3-1* mutant have fewer ribosomes. However, this does not appear to have a significant impact on the synthesis of mitochondrially encoded proteins, as the majority of these proteins are present in comparable abundance to the wild type (Figs. 3 and 5). Editing of 3 ribosomal transcripts is reduced in the *morf3-1* line, rps3 (ATMG00090), rpl5 (ATMG00210), and rps4 (ATMG00290). For rps4, peptides matching a differentially edited site (rps4eU299PLp60) were found (Table 1). In transcripts from mitochondria of wild-type plants, editing at this site is 60%; in the *morf3-1* line, editing could not be detected (Takenaka et al. 2012), further supporting the assumption that RNA editing frequency has to be drastically reduced for unedited proteoforms to accumulate in detectable amounts (van Wijk et al. 2024). In our study, only peptides matching the nonedited transcripts were found. To determine whether this proteoform is incorporated into mitochondrial ribosomes and whether it impairs their assembly or stability, a combination of large-pore blue-native PAGE (lpBN-PAGE) and crosslinking could be employed (Rugen et al. 2019, 2021).

For mitochondrial complexes III, IV, and V, we could not characterize the “editing status” of the corresponding mitochondrially encoded subunits. However, all 3 complexes seem to exhibit no to minimal changes in their abundance in the *morf3-1* mutant (Fig. 3). Additionally, BN-PAGE analysis demonstrated that the stability of complexes III and V remains essentially unchanged (Figs. 5 and 9). Only complex V might have a slightly reduced heat stability (Fig. 9). The editing frequencies of the subunits COB, COX3, and ORF25/ATP synthase subunit b in the *morf3-1* mutant line remain relatively high (50% to 80%). We conclude that MORF3-deficient mitochondria maintain a sufficiently large population of fully edited proteoforms. This conclusion is also confirmed by the fact that these subunits did not show any altered properties in our complexome profiling experiments (Supplementary Fig. S7).

In summary, we conclude that the observed phenotype of the *morf3-1* line is essentially based on the impaired complex I function. This is in agreement with results on other complex I mutants, for example, a mutant in which the splicing of Nad subunits is disturbed and in which the assembly of complex I was also found to be disturbed, resulting in a developmental delay of the mutant plants (Weißenberger et al. 2017).

Do these results indicate that complete editing of transcripts is a prerequisite for the full functionality of the mitochondria? Based on the proteomic data for the *morf3-*1 line, this seems to be true for complex I. However, some editing sites in other mitochondrial transcripts are only partially edited even in wild-type plants. It has been shown that varying proteoforms are translated from these transcripts and even incorporated into protein complexes, such as the small mitoribosomal subunit (Rugen et al. 2024). Proteins resulting from differential editing may well have differential physiological functions. This hypothesis should be further investigated.

## Materials and methods

### Plant material

A homozygous T-DNA insertion line (GK-109E12.01) carrying a T-DNA insertion at the gene locus AT3G06790 (encoding MORF3) and a corresponding wild-type *Arabidopsis* (*Arabidopsis thaliana*) Col-0 line were acquired from the Nottingham *Arabidopsis* Stock Centre (NASC). Seeds were stratified for 2 d at 4 °C. Plants were grown in a climate chamber under long day conditions (16 h light, 8 h dark, 22 °C, 120 µmol s^−1^m^−2^ light, 55% humidity). After 2 weeks, homozygous plants with respect to the T-DNA insertions were confirmed by PCR genotyping using gene-specific primers (5′-CGAGGACGAAATTTTCTTTCTG-3′ and 5′-GAACACCAGGCAATGCTAATG-3′) and a left border primer for the T-DNA insertion (5′-ATATTGACCATCATACTCATTGC-3′). For root growth experiments, plants were grown vertically on agar plates containing 2.15 g/l Murashige and Skoog basal salt mixture, 0.5 g/l MES, 10 g/l sucrose, and 1 ml/l Gamborg’s vitamin solution. Plates were scanned after 10 d, and root lengths were determined using the ImageJ software package. Heterotrophic liquid cell cultures of the wild-type and the mutant *Arabidopsis* lines were established as described previously (Werhahn et al. 2001). Cells were transferred into fresh medium on a weekly basis.

### Isolation of mitochondria

Mitochondria were isolated from cell cultures 7 d after subcultivation. Isolation was achieved through differential centrifugations and Percoll density gradient centrifugation as described previously (Werhahn et al. 2001). Mitochondria from density gradients were subjected to 4 washing steps for Percoll removal. The concentration of the mitochondrial suspension was adjusted to 2.5 µg protein/µl using the Bradford assay (Thermo Fisher Scientific) on a plate reader according to the manufacturer's instructions. For all experiments (see below), individual isolates were used as biological replicates. For complexome profiling experiments, freshly isolated mitochondria were used, and BN-PAGE was performed directly after the determination of protein content.

### Protein extraction, digestion, and peptide clean-up for shotgun proteomics

Shotgun proteome analyses for both mitochondrial fractions were carried out in biological triplicates. Mitochondrial proteins were prepared for mass spectrometry analysis via the single-pot, solid-phase enhanced sample preparation (SP3) protocol developed by Hughes et al. (2019). Here, we used a protocol from Mikulášek et al. (2021) with minor adaptations: Mitochondrial isolates were each mixed with an equal volume of 2× SDT (SDS-DTT-Tris) buffer (8% [w/v] SDS [sodium dodecyl sulfate], 0.2 M DTT (dithiothreitol), 0.2% Tris-HCL, pH 7.6) and incubated on a thermal shaker (TS-100, Kisker Biotech, Steinfurt, Germany) for 1 h at 60 °C and 1,000 rpm. After centrifugation for 10 min at 20,000 × *g*, supernatants were transferred into new reaction tubes, sonicated in a water bath for 10 min (Elmasonic S30, Elma, Singen, Germany), and centrifuged again for 10 min at 20,000 × *g*. From the supernatant, a volume corresponding to 100 µg protein was transferred into a new reaction tube, and proteins were alkylated via incubation with 20 mM iodoacetamide (IAA) for 30 min at 600 rpm at room temperature in the dark. Alkylation was stopped by the addition of 5 mM DTT.

Sera-Mag bead carboxylate-modified hydrophilic solids (Global Life Sciences, Little Chalfont, Buckinghamshire, United Kingdom) were combined 1:1 with hydrophobic solids, and a total amount of 600 µg beads was added to each sample. Proteins were precipitated by addition of 70 µl ethanol (100%) and subsequent incubation for 10 min at 1,000 rpm and 24 °C. Beads were pelleted on a magnetic rack for 2 min, and proteins were washed 3 times with 140 µl of freshly prepared 80% ethanol. After protein clean-up, beads were transferred in fresh 80% ethanol into low protein-binding tubes (Low Binding Micro Tubes, Sarstedt, Nümbrecht, Germany), and ethanol was removed.

Proteins were digested with 2 µg of trypsin (V5117, sequencing grade, modified, Promega, Madison, Wisconsin, United States) in 50 mM ammonium bicarbonate (pH 7.8) at 37 °C and 1,000 rpm overnight in a total reaction volume of 60 µl. On the next day, protease activity was stopped by addition of 1% (v/v) formic acid (FA). The pH of each sample was controlled and adjusted to < 3.

Peptides were directly cleaned up via solid-phase extraction on Sep-Pak Vac 1 cc (50 mg) tC18 cartridges (Waters, Eschborn, Germany). Cartridges were wetted with 1 ml 100% acetonitrile and 1 ml 0.1% (v/v) formic acid in 50% (v/v) acetonitrile. Cartridge equilibration was performed by adding 2 × 1 ml of 0.1% FA (v/v) in H_2_0. Acidified peptides (pH < 3) were loaded onto the cartridges, washed 2 times with 0.1% FA (v/v) in H_2_O, and eluted twice in 200 µl of 0.1% FA (v/v) in 50% (v/v) acetonitrile (ACN). Cleaned peptides were dried in a vacuum centrifuge and stored at −20 °C. Final peptide concentration was determined with the Pierce peptide quantification kit (Thermo Fisher Scientific, Bremen, Germany) following the manufacturer’s instructions.

### LC-IMS-MS/MS analysis (shotgun experiments)

A nanoElute2 UHPLC (Bruker Daltonics, Bremen, Germany) was coupled to a timsTOF Pro ion mobility spectrometry quadrupole time of flight mass spectrometer (Bruker Daltonics, Bremen, Germany). Peptides (200 ng) reconstituted in 0.1% FA were directly transferred onto an “Aurora” reversed phase analytical column with integrated emitter tip (25 cm × 75 μm inner diameter, IonOpticks, Fitzroy, Australia). Peptides were separated on the analytical column at 50 °C via a 70 min gradient at a flow rate of 300 nl min^−1^. A linear gradient composed of solvent A (0.1% FA in LC-MS grade water) and solvent B (0.1% FA in 100% ACN, LC-MS grade) from 2% B to 37% B for the first 60 min was followed by a 10 min washing step at 95% B.

The timsTOF Pro mass spectrometer was operated in DDA-PASEF mode. Automatic recalibration of ion mobility (IM) before each sample run was activated. Each sample was measured 3 times with different ion mobility windows: MS and MS/MS scan range was 100 to 1,700 m/z, the ion mobility ranges (1/K_0_) for the 3-range peptide IM fractionation were 0.7 to 1.1 V×s/cm^2^, 0.9 to 1.2 V×s/cm^2^, and 1.1 to 1.4 V×s/cm^2^ according to Guergues et al. (2022). A polygon filtering was applied in the m/z and ion mobility area to exclude the low m/z of singly charged ions for PASEF precursor selection. Ramp and accumulation time was set to 100 ms to achieve close to 100% duty cycle. The number of PASEF ramps was set to 10 with a charge maximum of 5. The quadrupole isolation width was set to 2 for m/z = 700 and 3 for m/z = 800. Collision energy was 20 eV for ion mobility (1/K_0_) 0.6 V×s/cm^2^ and 59 eV for ion mobility (1/K_0_) 1.6 V×s/cm^2^, respectively.

### Database searches (shotgun experiments)

FragPipe (version 22; Yu et al. 2020) was used to query acquired MS/MS spectra against a modified in-house TAIR10 + Araport11 database including permutation variants of mitochondrially encoded sequences to search for RNA editing-specific proteoforms (Rugen et al. 2024). Decoy sequences were generated and appended to the original database for MSFragger as well as sequences of common contaminants. In FragPipe, the preconfigured workflow “LFQ_MBR” was selected. Peptide sequence identification was performed with MS Fragger version 4.1 (Kong et al. 2017). Precursor and fragment mass tolerance was set to 20 ppm, and mass calibration and parameter optimization were enabled. Tryptic cleavage specificity with up to 2 missed-cleavage sites was applied, along with variable methionine oxidation, variable protein N-terminal acetylation, and fixed carbamidomethyl cysteine modifications. The allowed peptide length and mass ranges were 7 to 50 residues and 500 to 5000 Da, respectively. Peptide-to-spectrum matches (PSMs) were rescored by MSBooster (version 1.2.31) (Yang et al. 2023) and Percolator (version 3.6.5) (Käll et al. 2007) using retention time and MS/MS spectra predictions generated by AlphaPeptDeep (Zeng et al. 2022). Based on PSMs, identified proteins were inferred by ProteinProphet (Nesvizhskii et al. 2003). Philosopher (version 5.1.1) was used to perform FDR filtering (1% FDR at the PSM, ion, peptide, and protein level). Quantification analysis was performed with IonQuant (version 1.10.27; Yu et al. 2021) using default parameters. “Protein intensities” and “MaxLFQ intensities” were calculated via the top-N (Yu et al. 2020, 2021) or the MaxLFQ method (Cox et al. 2014; Yu et al. 2021), respectively. Match between runs (MBR) was enabled with an MBR ion FDR of 1%.

### Differential expression analysis

Shotgun proteomic data were statistically analyzed using Perseus (version 1.6.15.0; Tyanova and Cox 2018). Protein groups marked as “potential contaminant,” “only identified by site,” or “reverse” were removed prior to the primary analysis. Only those protein groups with a calculated MaxLFQ intensity greater than 0 in at least 4 out of 6 samples were considered for further analysis. Missing protein intensities were then considered as too low for proper quantification and replaced by very low values from a normal distribution. Log2​-transformed MaxLFQ intensities were then used to identify differentially expressed proteins in the *morf3-1* mutant.

### Manual inspection of editing-specific peptides

The “editing status” of a protein can be indirectly determined through “editing-specific” peptides. These peptides contain amino acids whose identity is altered by RNA editing and can exist in both “edited” and “unedited” forms. For the analysis of a protein editing status, we considered only those peptides with an Andromeda peptide score of at least 100 (complexome profiling data, see below) or a Hyperscore of at least 20 (shotgun data). Additionally, peptides were only included if the amino acid site affected by RNA editing was covered by either a b-ion or a y-ion in the MS/MS spectra. To prevent potential misinterpretations from “one-hit wonders,” we included peptides identified in at least 2 distinct MS/MS spectra. Furthermore, we did not accept editing specific peptides that were identified in a sample solely through “match between runs.” For each amino acid substitution resulting from partial editing, we examined whether a posttranslational modification (PTM) at the edited site could mimic a similar mass change. To assess this, we calculated the mass shift caused by the amino acid exchange and searched for PTMs with a similar mass change (±2 Da) using the UNIMOD platform (Creasy and Cottrell 2004). Spectra were manually inspected using PDV (Li et al. 2019).

### Blue-native polyacrylamide gel electrophoresis (BN-PAGE)

For BN-PAGE, freshly isolated mitochondria were resuspended in resuspension buffer (400 mM mannitol, 1 mM EGTA, 10 mM tricine, 0.2 mM PMSF, pH 7.4), and mitochondrial proteins were quantified via the Bradford assay. A volume corresponding to 250 µg of protein was mixed with an equal volume of digitonin solubilization buffer (30 mM HEPES, 150 mM potassium acetate, 10% [v/v] glycerol, 5% [w/v] digitonin; Klodmann et al. 2011) and incubated for 20 min on ice. A Coomassie solution (750 mM ACA, 5% [w/v] Coomassie G 250) was added to the samples to a concentration of 5% (v/v). BN-PAGE was carried out as described previously (Wittig et al. 2006). Samples were separated in 4.5% to 16% polyacrylamide gradient gels, first, for 45 min at 7 mA and 100 V followed by 13 h at 15 mA and max. 500 V. The resulting gels were stained either with Coomassie Brilliant Blue or by NADH dehydrogenase activity staining according to Jung et al. (2000).

For the comparison of digitonin and n-dodecyl-β-D-maltoside (DDM), both detergents were used to solubilize membrane proteins from pelleted and frozen mitochondrial samples corresponding to 150 µg of protein, and a subsequent BN-PAGE of all samples was performed on the same gel. Samples were treated as described above with the following exceptions: For digitonin solubilization, pellets were resuspended in digitonin solubilization buffer (2.5% [w/v] digitonin, 30 mM HEPES, 150 mM potassium acetate, 10% [v/v] glycerol), and for DDM solubilization, pellets were resuspended in DDM solubilization buffer (1.6% [w/v] DDM, 750 mM aminocaproic acid, 50 mM Bis-Tris, 0.5 mM EDTA, pH 7.0). Samples were incubated for 20 min on ice.

### Investigation of the thermal stability of OXPHOS complexes

Starting material for thermal stability experiments was 3 independent mitochondrial isolates from *Arabidopsis* wild-type and *morf3-1* mutant lines. Freshly isolated organelles were resuspended in resuspension buffer (400 mM mannitol, 1 mM EGTA, 10 mM tricine, 0.2 mM PMSF, pH 7.4), and protein concentrations were quantified using the Bradford assay (Thermo Fisher Scientific) on a plate reader according to the manufacturer's instructions. Mitochondria equivalent to 450 µg of protein were aliquoted, and the volume of each sample was subsequently adjusted to 210 µl using resuspension buffer. Organelle fractions from wild-type and *morf3-1* mutant lines were incubated in parallel for 30 min on ice, at 22 °C or at 37 °C, respectively. After incubation, 210 µl of digitonin solubilization buffer (30 mM HEPES, 150 mM potassium acetate, 10% [v/v] glycerol, 5% [w/v] digitonin) was added to the mitochondria and incubated for a further 20 min on ice. The samples were centrifuged at 18,300 × *g* for 10 min, and volumes equivalent to 150 µg of protein of each sample were mixed with a Coomassie solution to a concentration of 5% (v/v). Samples were separated in 4.5% to 16% polyacrylamide gradient gels first, for 45 min at 7 mA and 100 V followed by 13 h at 15 mA and max. 500 V. The resulting gels were stained with Coomassie Brilliant Blue. Gels were scanned at high resolution and imported into the Delta2D software package (Decodon, version 4.5.3). Bands were detected by the automatic mode of the software package and adjusted manually. The band volumes were quantified by Delta2D as the product of intensity and area.

### Complexome profiling experiments

Coomassie-stained BN gel lanes were cut into 43 fractions from bottom to top. Subsequently, each fraction was subjected to in-gel trypsin digestion and peptide extraction: Gel pieces were dehydrated using 100% ACN. For cysteine alkylation, gel pieces were first incubated with 20 mM DTT for 30 min at 56.8 °C, dehydrated with 100% ACN, and incubated in 55 mM IAA at room temperature for the same time in the dark. The dehydrated gel pieces were incubated in 0.1 M ammonium bicarbonate, and digestion was performed for 18 h at 37.8 °C using trypsin (V5117, sequencing grade, modified, Promega, Madison, Wisconsin, United States) at a concentration of 2 mg/mL in 0.1 M ammonium bicarbonate. Tryptic peptides were extracted by incubation with 5% FA in 50% ACN for 20 min at 37 °C. Supernatants were collected, and peptides were further extracted with 1% FA in 50% ACN for 20 min at 37 °C. The supernatants were combined with the initial supernatant of each sample, respectively. The final extraction step involved incubating the gel pieces in 100% ACN for 20 min at 37 °C. The supernatant was then pooled with both the first and second supernatants. The extracted peptides were subsequently dried using vacuum centrifugation and stored at −20 °C.

Peptides were dissolved in 20 µl sample solution (5% [v/v] ACN, 0.1% FA in H2O), and 5 µl of peptide solution was loaded into a liquid chromatography system (Thermo Fisher Scientific). Samples were separated on a 50 cm C18 reversed phase analytical column (Acclaim PepMap 100, diameter 75 µm, pore size 100 Å; Thermo Fisher Scientific). The peptides were eluted at a flow rate of 250 nl min^−1^ using a gradient of ACN, increasing from 5% to 36% over a time period of 60 min at 45 °C column temperature. Eluting peptides were transferred into the mass spectrometer (Q Exactive, Thermo Fisher Scientific) by electrospray ionization (ESI) using a stainless-steel nano-bore emitter attached to an NSI source. The spray was set to 2.2 kV and the capillary temperature to 275 °C. The MS was set to positive ion mode, and MS/MS spectra (Top10) were recorded from 20 to 100 min. For full MS, the scan range was set to 400 to 1600 m/z at a resolution of 70,000. For dd-MS^2^ scans, the resolution was set to 17,000. Automatic gain control (AGC) targets were set to 1e6 and 1e5.

### Database search (complexome profiling experiments)

MaxQuant (version 2.0.3.0; Tyanova et al. 2016) was used to query acquired MS/MS spectra against a modified in-house TAIR10 + Araport11 database including permutation variants of mitochondrially encoded sequences to search for RNA editing-specific proteoforms (Rugen et al. 2024). Carbamidomethyl (C) was specified as a fixed modification, and oxidation (M) and acetylation (protein N-Term) were considered as variable modifications. Default parameters were used with the following exceptions: calculation of iBAQ (Schwanhäusser et al. 2011) values was activated, and the options “log fit” and “charge normalization” were enabled. Trypsin/P was set as the proteolytic enzyme with up to 2 missed-cleavage sites. The minimal peptide length was set to 7 amino acids, and the maximum peptide mass to 4,600 Da. Identification transfer between individual runs via the “match between runs” feature was enabled. The match time window was set to 0.7 min, and the alignment time window was set to 20 min. The FDR was 1% at both the PSM and protein levels.

### Generation of complexome maps

For building complexome maps, protein abundance profiles of complex I subunits, based on the iBAQ values calculated by MaxQuant, were sorted manually according to their appearance in the complex assembly described by Ligas et al. (2019). Sorted profiles were then individually visualized as heatmaps in the NOVA software (version 0.8.0, Giese et al. 2015). Wild-type and *morf3-1* mutant heat maps were then normalized and compared using the NOVA compare function. The wild-type heat map was selected as the reference for normalization. Values of the *morf3-1* heatmap were adjusted to the reference accordingly.

### High-resolution respirometry

Respiration rates of freshly isolated mitochondria from wild-type and *morf3-1* mutant *Arabidopsis thaliana* cell cultures were assessed using a high-resolution respirometry system (Oroboros Oxygraph-2k, Oroboros Instruments, Innsbruck, Austria) at 25 °C. Mitochondrial respiration buffer (300 mM sucrose, 5 mM KH_2_PO_4_, 10 mM TES, 10 mM NaCl, 2 mM MgSO_4_, 1% [w/v] BSA, pH 6,8) was added to the reaction chamber, and mitochondria were injected at a final concentration of 40 µg ml^−1^. To stimulate respiration, NAD^+^ (2 mM), sodium–pyruvate (10 mM), malate (10 mM), and ADP (300 µM) were sequentially added. Rotenone (5 µM) was introduced to inhibit complex I, followed by SHAM (20 mM) and KCN (1 mM) to inhibit O_2_ reduction by alternative oxidases and complex IV, respectively.

### Phylogenetic analysis of RNA editing sites

Phylogenetic data were retrieved from the NCBI taxonomy browser (Schoch et al. 2020). Information on the conservation of complex I RNA editing sites was retrieved from the PREPACT portal (Lenz et al. 2018). The phylogenetic tree was built with the FigTree software (version 1.4.4; http://tree.bio.ed.ac.uk/software/figtree).

### Structural modeling

The 3D structure of the *Arabidopsis* thaliana complex I was obtained from the Protein Data Bank (PDB). The dataset is identified by the ID 8BPX and was published by Klusch et al. (2023). The amino acid substitutions caused by incomplete RNA editing were manually introduced into the structure using the “swapaa” function in the software ChimeraX, version 1.9 (Meng et al. 2023).

### Accession numbers

Sequence data from this article can be found in the GenBank/EMBL data libraries under accession numbers:

BT002982.1 (MORF3; At3g06790); EF488917.1 (Nad4L; ATMG00650); X96535.1 (Nad2; ATMG00285); EF488914.1 (Nad3; ATMG00990); BK010421.1 (Nad4; ATMG00580); X60045.1 (Nad5; ATMG00513); EF488922.1 (Nad6; ATMG00270); BK010421.1 (Nad7; ATMG00510); AC007063 (SLO2; AT2G13600); EF488934.1 (TatC; ATMG00570); BT004558.1 (MNLL; AT4G16450). EF488944.1 (RPS4; ATMG00290).

## Supplementary Material

kiaf471_Supplementary_Data

## Data Availability

The raw mass spectrometry proteomics data as well as the MaxQuant and FragPipe analysis outputs have been deposited to the ProteomeXchange Consortium (http://proteomecentral.proteomexchange.org; Deutsch et al. 2023) via the Mass Spectrometry Interactive Virtual Environment (MassIVE) repository. The shotgun proteomics data are saved as a MassIVE dataset MSV000097179 and can be accessed following the link: https://massive.ucsd.edu/ProteoSAFe/dataset.jsp?accession=MSV000097179 (doi:10.25345/C55M62K4X ) . The complexome profiling data are saved as a MassIVE dataset MSV000097178 and can be accessed following the link: https://massive.ucsd.edu/ProteoSAFe/dataset.jsp?accession=MSV000097178 (doi:10.25345/C5988305R).

## Supplementary data

The following materials are available in the online version of this article.

Additional Supplementary data are provided with this manuscript:

**Supplementary Figure S1.** Experimental setup.

**Supplementary Figure S2.** Estimation of purity of the mitochondrial fractions isolated from *Arabidopsis* wild-type and *morf3-1* mutant lines.

**Supplementary Figure S3.** Abundance profiles of subunits of the complexes III, IV, and V along BN gel lanes in wild-type and *morf3-1* mutant *Arabidopsis* lines.

**Supplementary Figure S4.** Representative MS/MS spectra of peptides specific for RNA editing site nad2eU821SFp100 as detected by shotgun proteomics.

**Supplementary Figure S5.** Representative MS/MS spectra of peptides specific for RNA editing site nad7eU739LFp100 as detected by shotgun proteomics.

**Supplementary Figure S6.** Representative MS/MS spectra of peptides specific for RNA editing site nad2eU821SFp100 as detected by complexome profiling.

**Supplementary Figure S7.** Representative MS/MS spectra of peptides specific for RNA editing site nad7eU739LFp100 as detected by complexome profiling.

**Supplementary Figure S8.** Complex I assembly intermediates in wild-type and *morf3-1* mutant *Arabidopsis* plants as displayed by complexome profiling.

**Supplementary Figure S9.** Effects of disturbed Nad5 transcript editing on the structure of complex I in the *morf3-1* mutant line.

**Supplementary Figure S10.** Effects of disturbed Nad7 transcript editing on the structure of complex I in the *morf3-1* mutant line.

**Supplementary Figure S11.** Effects of disturbed Nad2 transcript editing on the structure of complex I in the *morf3-1* mutant line.

**Supplementary Figure S12.** Impact of solubilization conditions on the visibility of OXPHOS complexes from wild-type and morf3-1 mutant lines upon their separation by 1D blue-native PAGE.

**Supplementary Figure S13.** Effects of disturbed Nad1 transcript editing on the structure of complex I in the *morf3-1* mutant line.

**Supplementary Table S1.** Overview of all proteins identified in this study.

**Supplementary Table S2.** Overview of all mitochondrial RNA editing sites affected in the *morf 3-1* mutant.

**Supplementary Table S3.** Summary of amino acid changes in complex I subunits caused by the *morf3-1* mutation.

**Supplementary Table S4.** Overview of all complexomes in this publication, displayed in a color-blind friendly palette.
