# Supplementary material for: Disturbed RNA editing in MORF3-deficient Arabidopsis mitochondria leads to impaired assembly of complex I
Source: Plant Physiol. 2025 Sep 30;199(2):kiaf471. doi: 10.1093/plphys/kiaf471 (PMC12541391; doi:10.1093/plphys/kiaf471)
Supplement: kiaf471_Supplementary_Data [file kiaf471_supplementary_data.zip › Supplementary_Data.pdf]

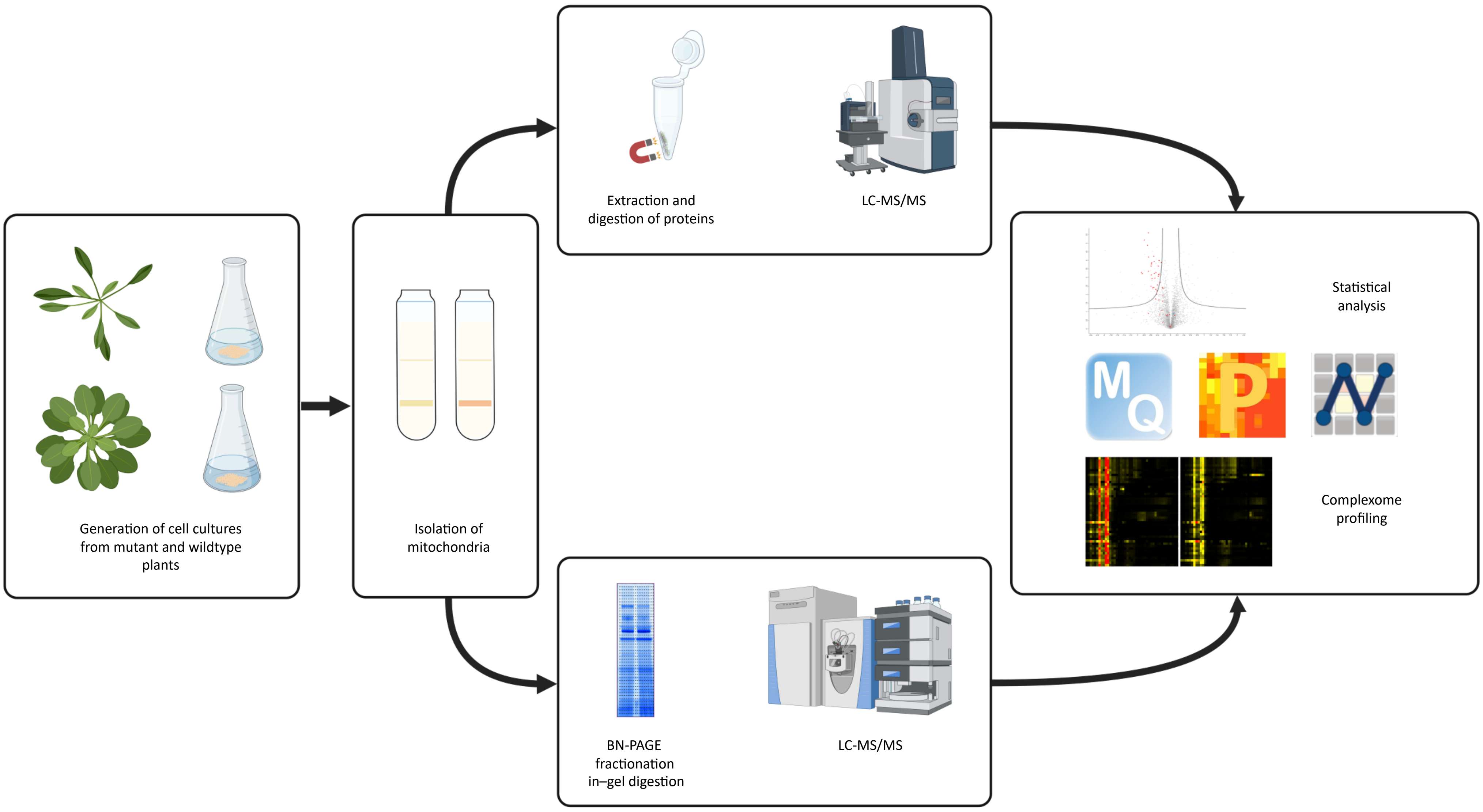

Supplementary Figure S1: Experimental set-up. Left: Arabidopsis wild-type and *morf3-1* mutant plant lines are used to establish cell cultures of the two lines. Cell cultures are used as starting material for mitochondrial isolations. Top: mitochondrial fractions are used for shotgun-protoeme analyses using a timsTOF II mass spectrometer (Bruker, Germany). Bottom: Alternatively, mitochondrial fractions are used for complexome profiling experiments by Blue-Native PAGE and subsequent shotgun analyses using a Q-exactive orbitrap mass spectrometer (Thermo, Germany). Right: Results form both experimental lines are integrated for in-depth conclusions on the proteomes of the two mitochondrial fractions.

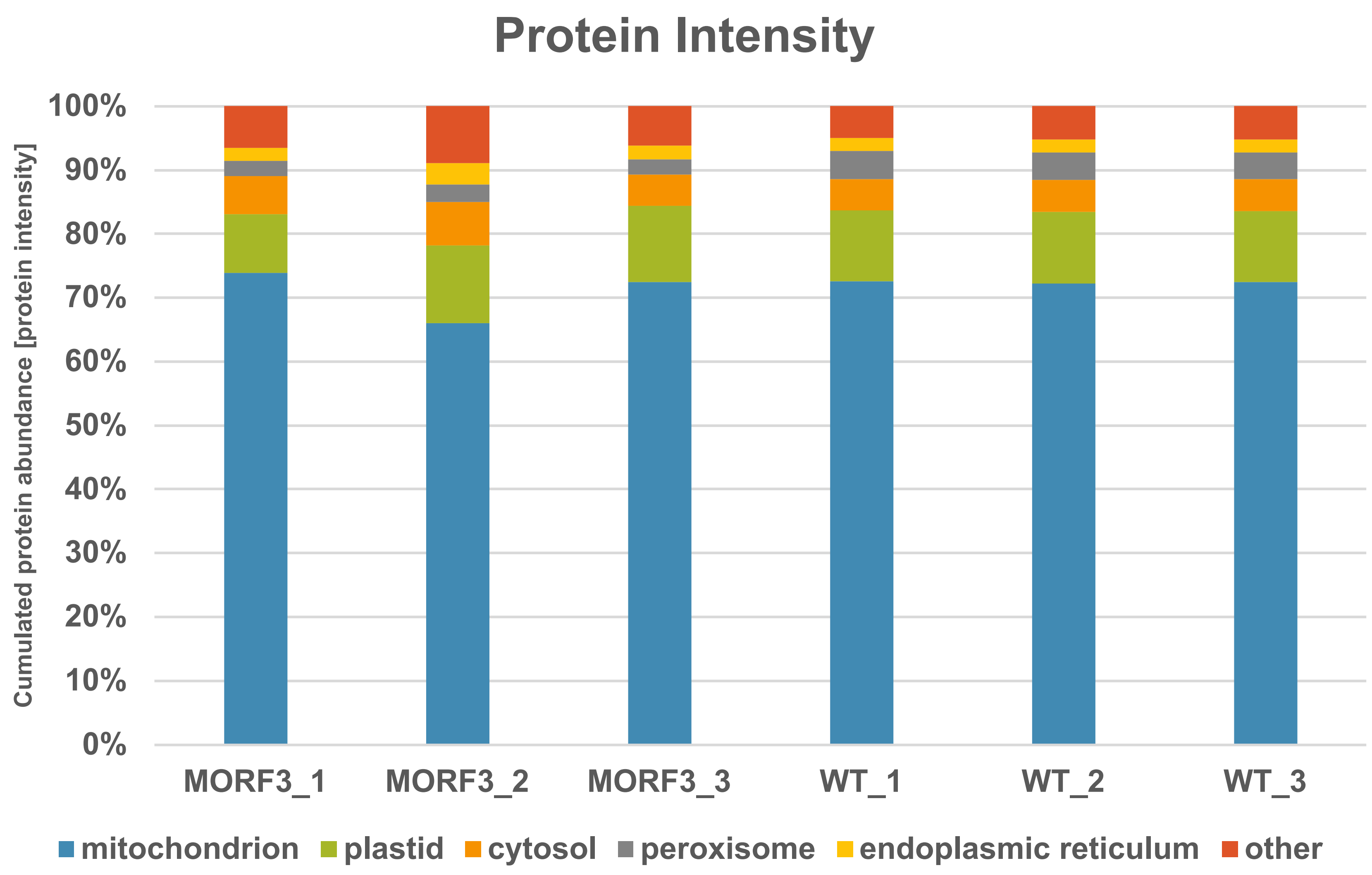

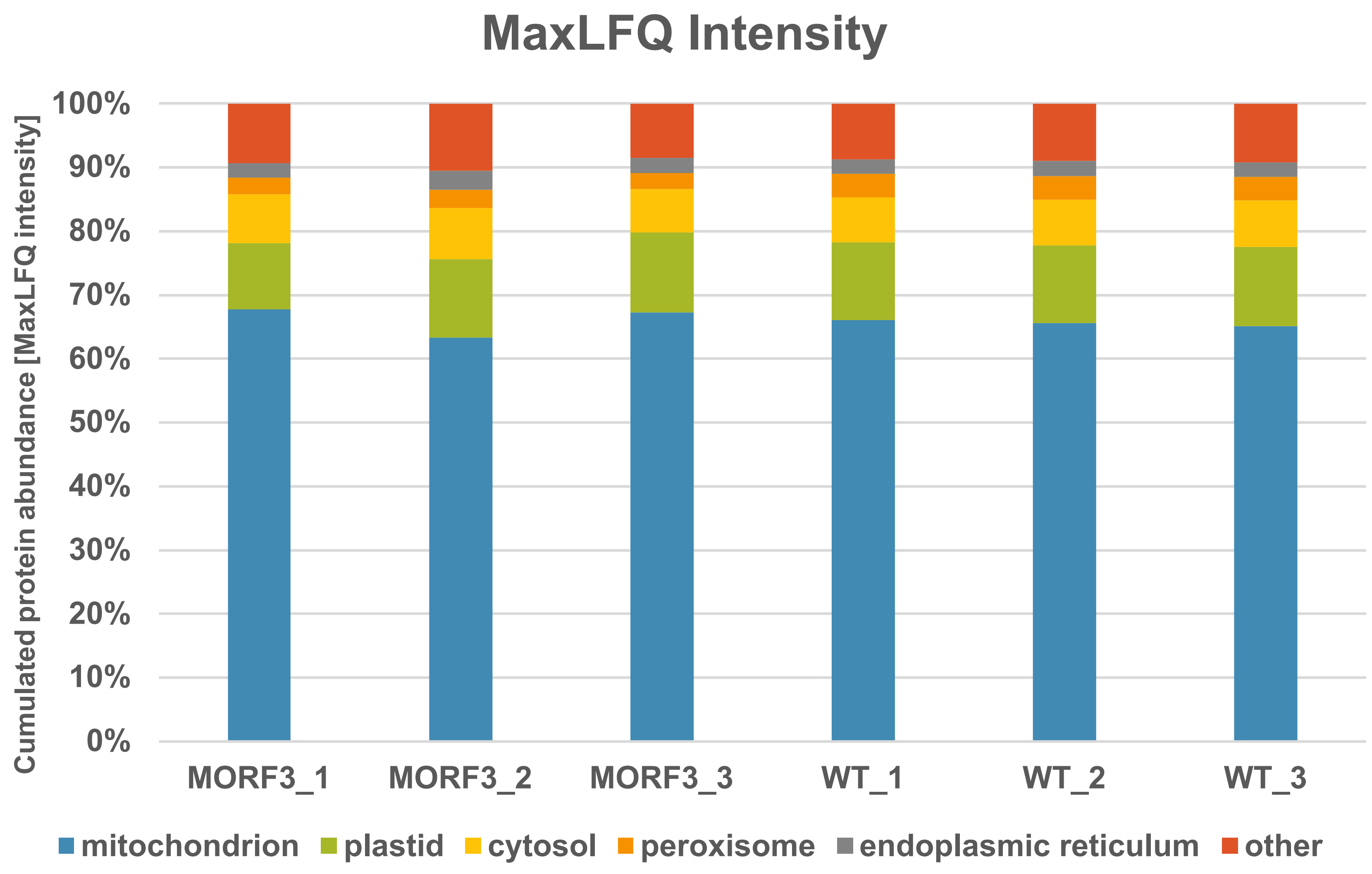

Supplementary Figure S2: Estimation of purity of the mitochondrial fractions isolated from Arabidopsis wild-type and *morf3-1* mutant lines. All proteins identified in the two mitochondrial fractions were assigned to subcellular localizations using the SUBAcon algorithm of SUBA5 (Hooper et al. 2017). Next, protein quantities (based on protein intensity calculations in FragPipe using the top-N method) were summed per subcellular fraction. The bar chart indicates cumulated protein abundance of the five most prominent subcellular compartments (mitochondrion, plastid, cytosol, peroxisome endoplasmatic reticulum; all other compartments are grouped as “others”) for three independent mitochondrial isolations from the Arabidopsis wild-type and *morf3-1* mutant lines. WT = wild type.

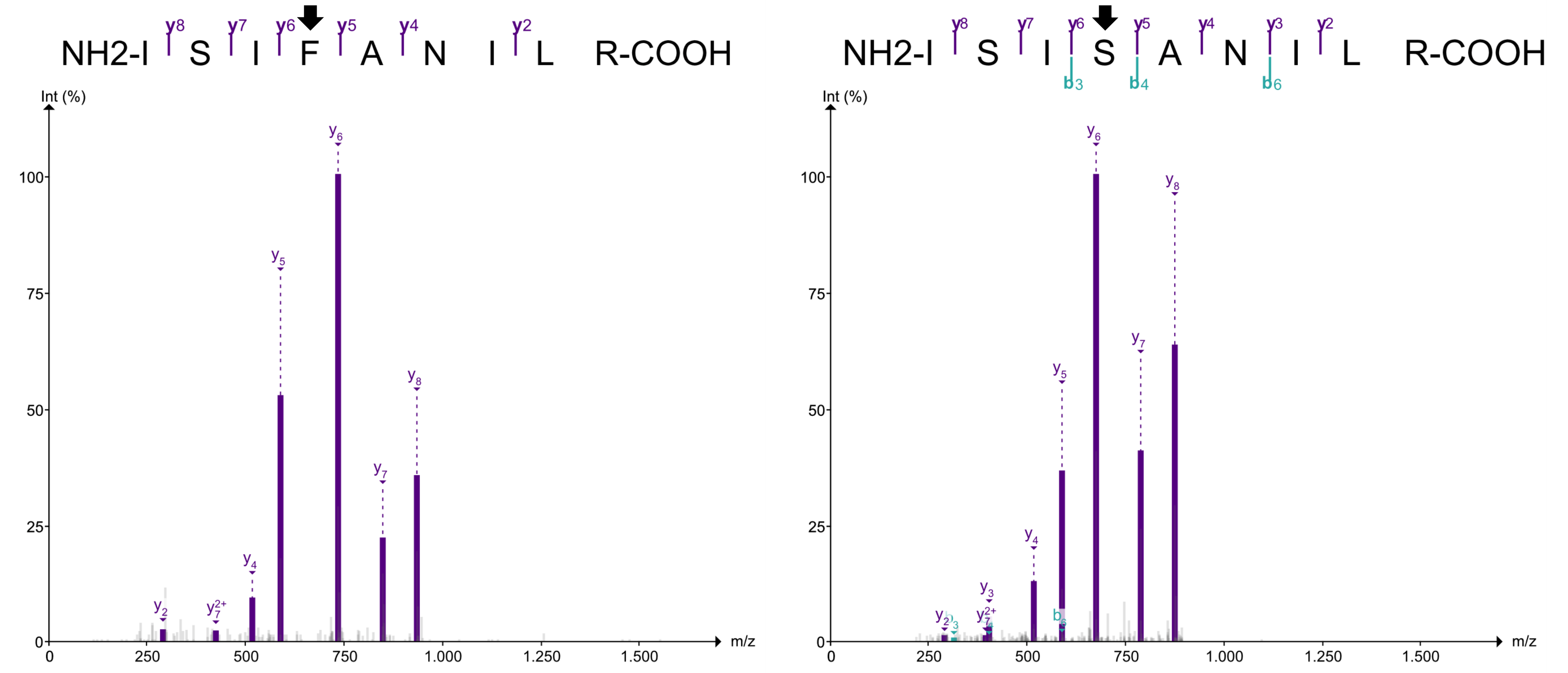

Peptide ISIFANILR

b	b++	#	Sequence	#	y	y++
114.09134	57.5493085	1	I	9		
201.123369	101.065323	2	S	8	933.551628	467.279452
314.207433	157.607355	3	I	7	846.519599	423.763438
461.275847	231.141562	4	F	6	733.435535	367.221406
532.312961	266.660118	5	A	5	586.367121	293.687199
646.355888	323.681582	6	N	4	515.330008	258.168642
759.439952	380.223614	7	I	3	401.28708	201.147178
872.524016	436.765646	8	L	2	288.203016	144.605146
		9	R	1	175.118952	88.0631143

Peptide ISISANILR

b	b++	#	Sequence	#	y	y++
114.09134	57.5493085	1	I	9		
201.123369	101.065323	2	S	8	873.515242	437.261259
314.207433	157.607355	3	I	7	786.483214	393.745245
401.239461	201.123369	4	S	6	673.39915	337.203213
472.276575	236.641926	5	A	5	586.367121	293.687199
586.319502	293.663389	6	N	4	515.330008	258.168642
699.403566	350.205421	7	I	3	401.28708	201.147178
812.48763	406.747453	8	L	2	288.203016	144.605146
		9	R	1	175.118952	88.0631143

monoisotopic mass S	87.0320284
monoisotopic mass F	147.068414
difference monoisotopic mass	60.0363855

Supplementary Figure S3: Representative MS/MS spectra of peptides specific for RNA editing site nad2eU821SFp100 as detected by shotgun proteomics. Annotated MS/MS spectra of RNA editing specific peptides. The peptide sequences are given above the spectra. Black arrows indicate the amino acid position affected by RNA editing. The y-axis shows the relative intensity of the peaks to each other. The x-axis indicates the mass-to-charge (m/z) ratio of each peak. Bars in purple represent y ions. Turquoise bars represent b ions. Gray bars indicate non-annotated peaks.

The peptide sequence and the positions of the respective b and y ions are shown at the center of the ion tables below each spectrum. For both peptides, the m/z value for the respective single and double (++) charged b (left) or y ions (right) is displayed. Detected ions are labeled in turquoise (b ions) or purple (y ions). The amino acid position affected by RNA editing is labeled in blue (edited) or red (unedited), respectively. The mass difference of the affected amino acids is calculated below the two ion tables.

Please note that the edited peptide (ISIFANILR) was detected in only one replicate of the *morf3-1* mutant and thus does not meet our filtering criteria. Both spectra were detected in the same sample.

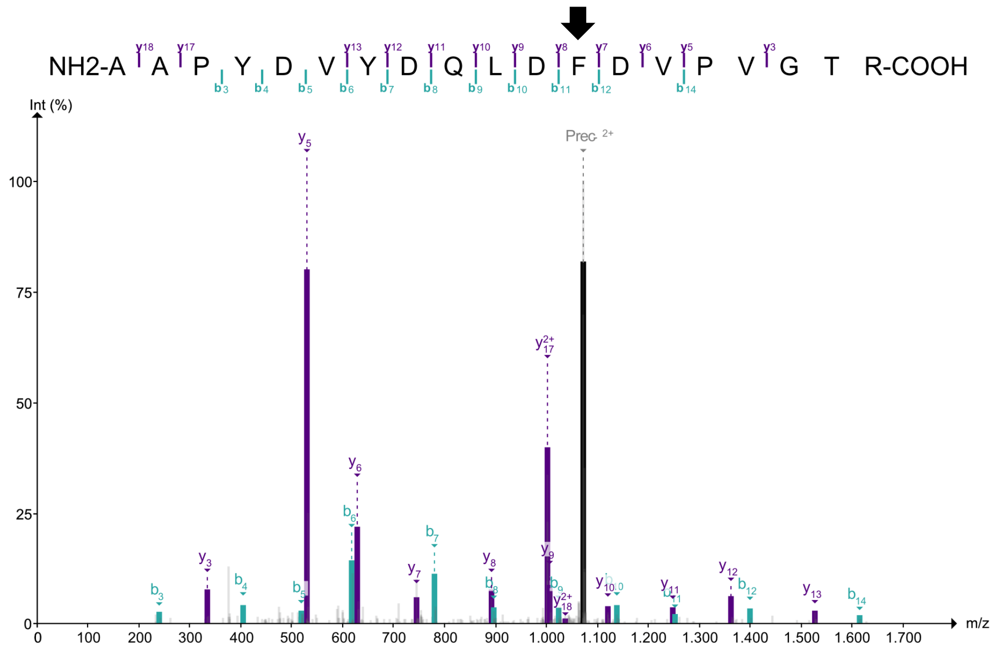

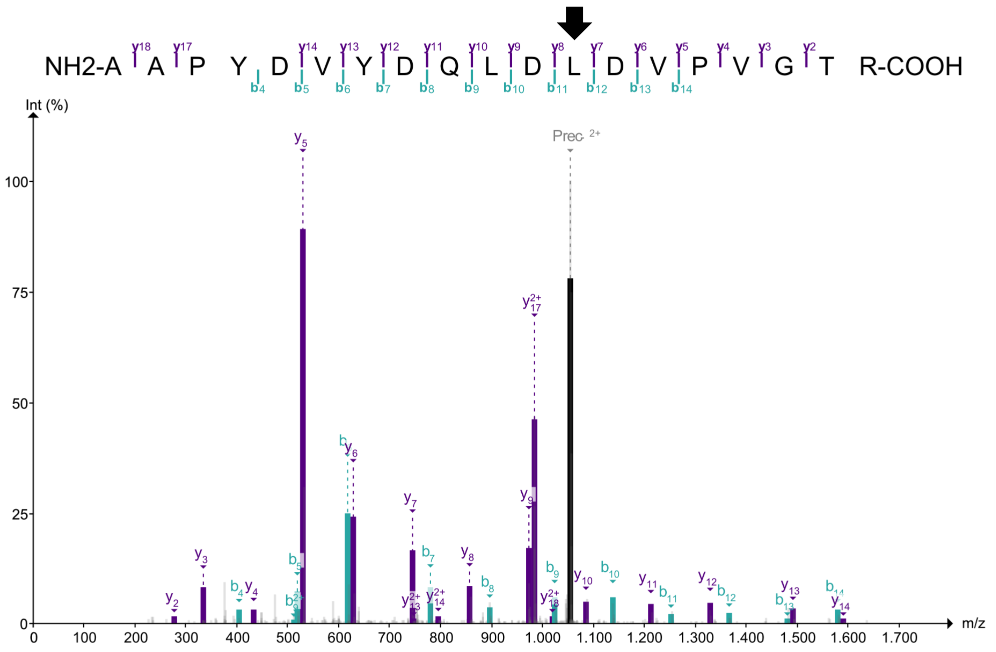

Peptide AAPYDVYDQLD FDVPVGTR

b	b++	#	Sequence	#	y	y++
72.0443903	36.5258334	1	A	19		
143.081504	72.0443903	2	A	18	2069.98146	1035.49437
240.134268	120.570772	3	P	17	1998.94435	999.975812
403.197596	202.102436	4	Y	16	1901.89158	951.44943
518.224539	259.615908	5	D	15	1738.82826	869.917766
617.292953	309.150115	6	V	14	1623.80131	812.404295
780.356282	390.681779	7	Y	13	1524.7329	762.870088
895.383225	448.195251	8	D	12	1361.66957	681.338423
1023.4418	512.224539	9	Q	11	1246.64263	623.824952
1136.52587	568.766571	10	L	10	1118.58405	559.795663
1251.55281	626.280043	11	D	9	1005.49999	503.253631
1398.62122	699.81425	12	F	8	890.473043	445.74016
1513.64817	757.327721	13	D	7	743.404629	372.205953
1612.71658	806.861928	14	V	6	628.377686	314.692481
1709.76934	855.38831	15	P	5	529.309272	265.158274
1808.83776	904.922517	16	V	4	432.256508	216.631892
1865.85922	933.433249	17	G	3	333.188094	167.097685
1966.9069	983.957088	18	T	2	276.166631	138.586954
		19	R	1	175.118952	88.0631143

Peptide AAPYDVYDQLD LDVPVGTR

b	b++	#	Sequence	#	y	y++
72.0443903	36.5258334	1	A	19		
143.081504	72.0443903	2	A	18	2035.99711	1018.50219
240.134268	120.570772	3	P	17	1964.96	982.983637
403.197596	202.102436	4	Y	16	1867.90723	934.457256
518.224539	259.615908	5	D	15	1704.84391	852.925591
617.292953	309.150115	6	V	14	1589.81696	795.41212
780.356282	390.681779	7	Y	13	1490.74855	745.877913
895.383225	448.195251	8	D	12	1327.68522	664.346249
1023.4418	512.224539	9	Q	11	1212.65828	606.832777
1136.52587	568.766571	10	L	10	1084.5997	542.803488
1251.55281	626.280043	11	D	9	971.515636	486.261456
1364.63687	682.822075	12	L	8	856.488693	428.747985
1479.66382	740.335546	13	D	7	743.404629	372.205953
1578.73223	789.869753	14	V	6	628.377686	314.692481
1675.78499	838.396135	15	P	5	529.309272	265.158274
1774.85341	887.930342	16	V	4	432.256508	216.631892
1831.87487	916.441074	17	G	3	333.188094	167.097685
1932.92255	966.964913	18	T	2	276.166631	138.586954
		19	R	1	175.118952	88.0631143

monoisotopic mass L	113.084064
monoisotopic mass F	147.068414
difference monoisotopic mass	33.9843499

Supplementary Figure S4: Representative MS/MS spectra of peptides specific for RNA editing site nad7eU739LFp100 as detected by shotgun proteomics. Annotated MS/MS spectra of RNA editing specific peptides. The peptide sequences are given above the spectra. Black arrows indicate the amino acid position affected by RNA editing. The y-axis shows the relative intensity of the peaks to each other. The x-axis indicates the mass-to-charge (m/z) ratio of each peak. Bars in purple represent y ions. Turquoise bars represent b ions. Gray bars indicate non-annotated peaks.

The peptide sequence and the positions of the respective b and y ions are shown at the center of the ion tables below each spectrum. For both peptides, the m/z value for the respective single and double (++) charged b (left) or y ions (right) is displayed. Detected ions are labeled in turquoise (b ions) or purple (y ions). The amino acid position affected by RNA editing is labeled in blue (edited) or red (unedited), respectively. The mass difference of the affected amino acids is calculated below the two ion tables. Both spectra were detected in the same sample.

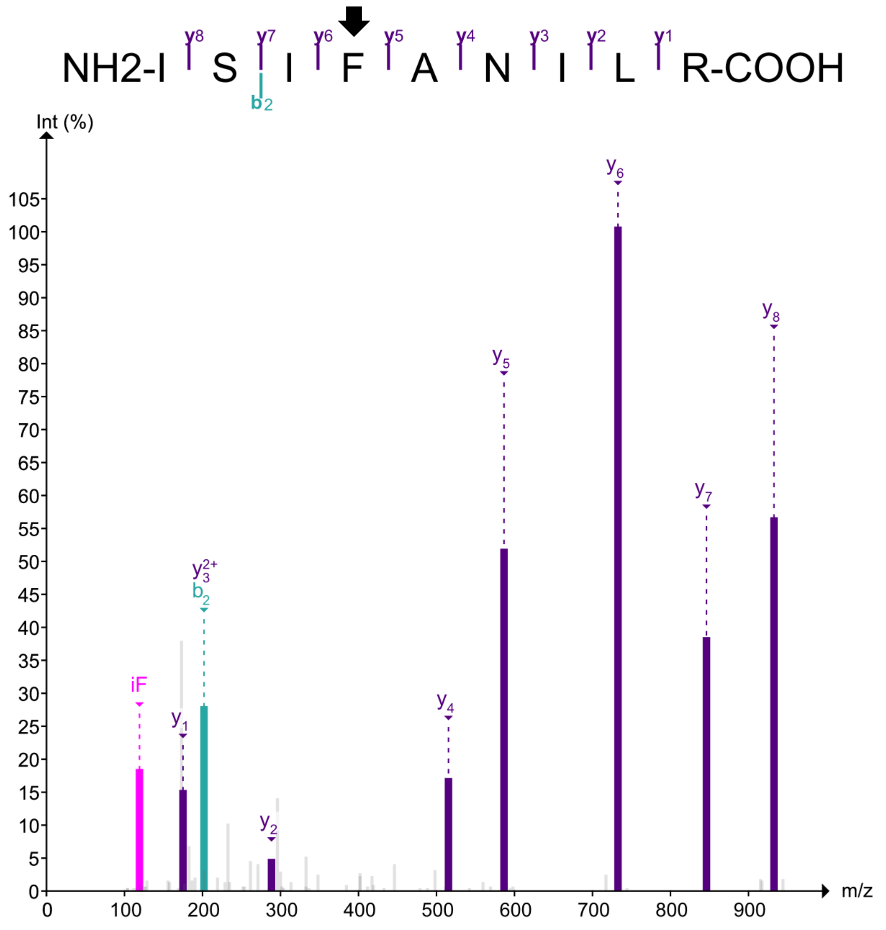

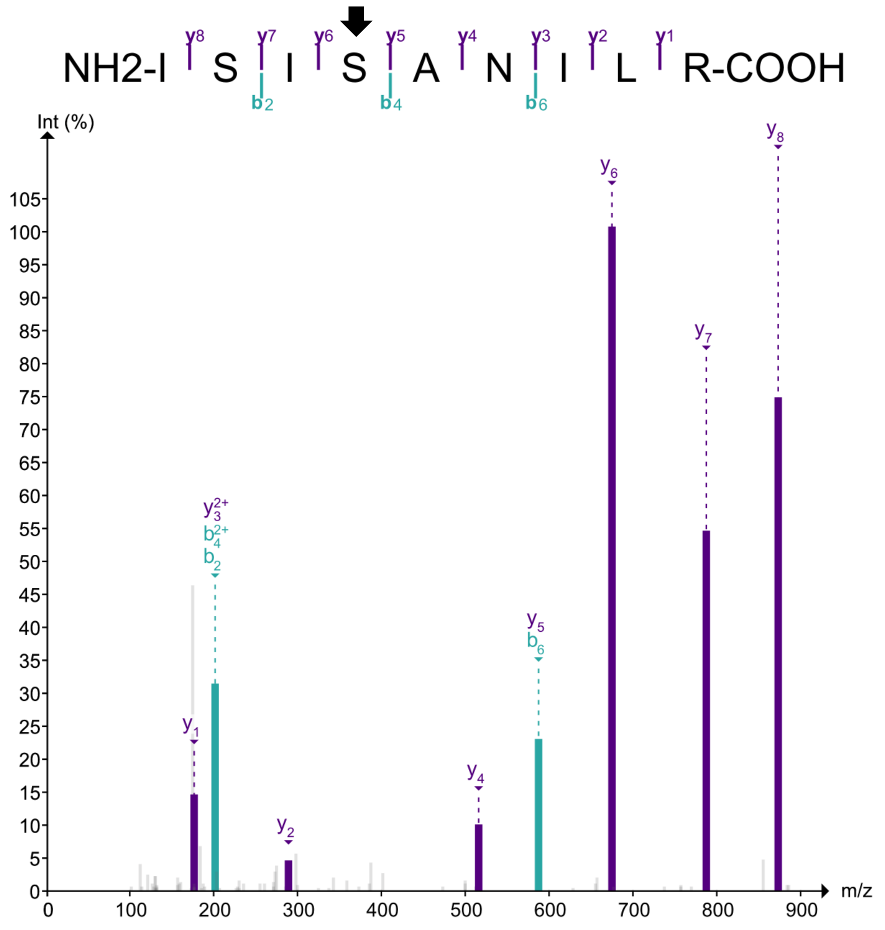

Peptide ISIFANILR

b	b++	#	Sequence	#	y	y++
114.09134	57.5493085	1	I	9		
201.123369	101.065323	2	S	8	933.551628	467.279452
314.207433	157.607355	3	I	7	846.519599	423.763438
461.275847	231.141562	4	F	6	733.435535	367.221406
532.312961	266.660118	5	A	5	586.367121	293.687199
646.355888	323.681582	6	N	4	515.330008	258.168642
759.439952	380.223614	7	I	3	401.28708	201.147178
872.524016	436.765646	8	L	2	288.203016	144.605146
		9	R	1	175.118952	88.0631143

Peptide ISISANILR

b	b++	#	Sequence	#	y	y++
114.09134	57.5493085	1	I	9		
201.123369	101.065323	2	S	8	873.515242	437.261259
314.207433	157.607355	3	I	7	786.483214	393.745245
401.239461	201.123369	4	S	6	673.39915	337.203213
472.276575	236.641926	5	A	5	586.367121	293.687199
586.319502	293.663389	6	N	4	515.330008	258.168642
699.403566	350.205421	7	I	3	401.28708	201.147178
812.48763	406.747453	8	L	2	288.203016	144.605146
		9	R	1	175.118952	88.0631143

monoisotopic mass S	87.0320284
monoisotopic mass F	147.068414
difference monoisotopic mass	60.0363855

Supplementary Figure S5: Representative MS/MS spectra of peptides specific for RNA editing site nad2eU821SFp100 as detected by complexome profiling. Annotated MS/MS spectra of RNA editing specific peptides. The peptide sequences are given above the spectra. Black arrows indicate the amino acid position affected by RNA editing. The y-axis shows the relative intensity of the peaks to each other. The x-axis indicates the mass-to-charge (m/z) ratio of each peak. Bars in purple represent y ions. Turquoise bars represent b ions. Gray bars indicate non-annotated peaks.

The peptide sequence and the positions of the respective b and y ions are shown at the center of the ion tables below each spectrum. For both peptides, the m/z value for the respective single and double (++) charged b (left) or y ions (right) is displayed. Detected ions are labeled in turquoise (b ions) or purple (y ions). The amino acid position affected by RNA editing is labeled in blue (edited) or red (unedited), respectively. The mass difference of the affected amino acids is calculated below the two ion tables.

Both spectra were detected in the same sample, which corresponds to fraction 33 in the *morf3-1* mutant complexome, where the monomeric complex I reaches its peak.

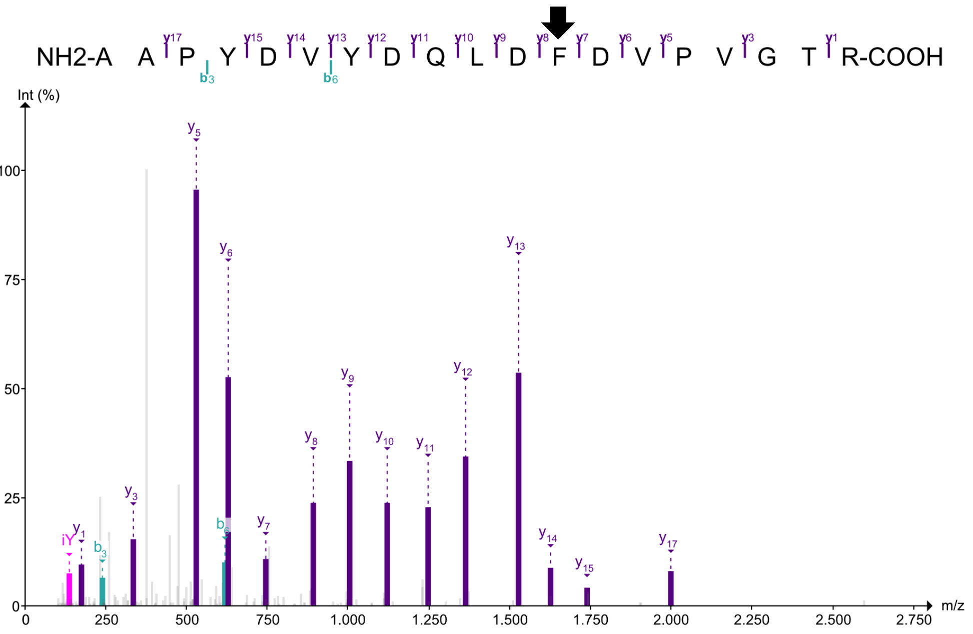

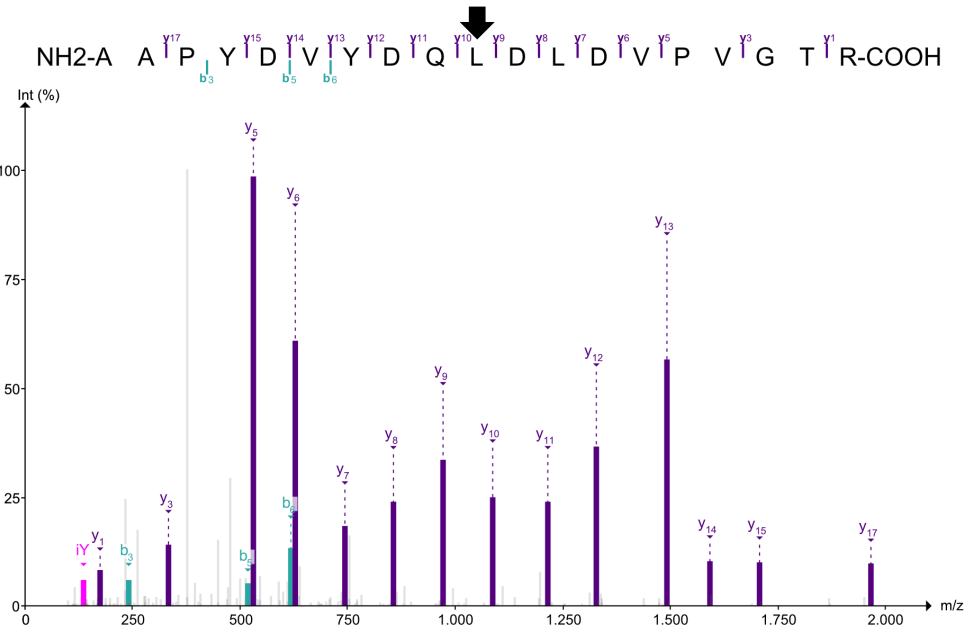

Peptide AAPYDVYDQLD FDVPVGTR

b	b++	#	Sequence	#	y	y++
72.0443903	36.5258334	1	A	19		
143.081504	72.0443903	2	A	18	2069.98146	1035.49437
240.134268	120.570772	3	P	17	1998.94435	999.975812
403.197596	202.102436	4	Y	16	1901.89158	951.44943
518.224539	259.615908	5	D	15	1738.82826	869.917766
617.292953	309.150115	6	V	14	1623.80131	812.404295
780.356282	390.681779	7	Y	13	1524.7329	762.870088
895.383225	448.195251	8	D	12	1361.66957	681.338423
1023.4418	512.224539	9	Q	11	1246.64263	623.824952
1136.52587	568.766571	10	L	10	1118.58405	559.795663
1251.55281	626.280043	11	D	9	1005.49999	503.253631
1398.62122	699.81425	12	F	8	890.473043	445.74016
1513.64817	757.327721	13	D	7	743.404629	372.205953
1612.71658	806.861928	14	V	6	628.377686	314.692481
1709.76934	855.38831	15	P	5	529.309272	265.158274
1808.83776	904.922517	16	V	4	432.256508	216.631892
1865.85922	933.433249	17	G	3	333.188094	167.097685
1966.9069	983.957088	18	T	2	276.166631	138.586954
		19	R	1	175.118952	88.0631143

Peptide AAPYDVYDQLD LDVPVGTR

b	b++	#	Sequence	#	y	y++
72.0443903	36.5258334	1	A	19		
143.081504	72.0443903	2	A	18	2035.99711	1018.50219
240.134268	120.570772	3	P	17	1964.96	982.983637
403.197596	202.102436	4	Y	16	1867.90723	934.457256
518.224539	259.615908	5	D	15	1704.84391	852.925591
617.292953	309.150115	6	V	14	1589.81696	795.41212
780.356282	390.681779	7	Y	13	1490.74855	745.877913
895.383225	448.195251	8	D	12	1327.68522	664.346249
1023.4418	512.224539	9	Q	11	1212.65828	606.832777
1136.52587	568.766571	10	L	10	1084.5997	542.803488
1251.55281	626.280043	11	D	9	971.515636	486.261456
1364.63687	682.822075	12	L	8	856.488693	428.747985
1479.66382	740.335546	13	D	7	743.404629	372.205953
1578.73223	789.869753	14	V	6	628.377686	314.692481
1675.78499	838.396135	15	P	5	529.309272	265.158274
1774.85341	887.930342	16	V	4	432.256508	216.631892
1831.87487	916.441074	17	G	3	333.188094	167.097685
1932.92255	966.964913	18	T	2	276.166631	138.586954
		19	R	1	175.118952	88.0631143

monoisotopic mass L	113.084064
monoisotopic mass F	147.068414
difference monoisotopic mass	33.9843499

Supplementary Figure S6: Representative MS/MS spectra of peptides specific for RNA editing site nad7eU739LFp100 as detected by complexome profiling. Annotated MS/MS spectra of RNA editing specific peptides. The peptide sequences are given above the spectra. Black arrows indicate the amino acid position affected by RNA editing. The y-axis shows the relative intensity of the peaks to each other. The x-axis indicates the mass-to-charge (m/z) ratio of each peak. Bars in purple represent y ions. Turquoise bars represent b ions. Gray bars indicate non-annotated peaks.

The peptide sequence and the positions of the respective b and y ions are shown at the center of the ion tables below each spectrum. For both peptides, the m/z value for the respective single and double (++) charged b (left) or y ions (right) is displayed. Detected ions are labeled in turquoise (b ions) or purple (y ions). The amino acid position affected by RNA editing is labeled in blue (edited) or red (unedited), respectively. The mass difference of the affected amino acids is calculated below the two ion tables.

Both spectra were detected in the same sample, which corresponds to fraction 33 in the *morf3-1* mutant complexome, where the monomeric complex I reaches its peak.

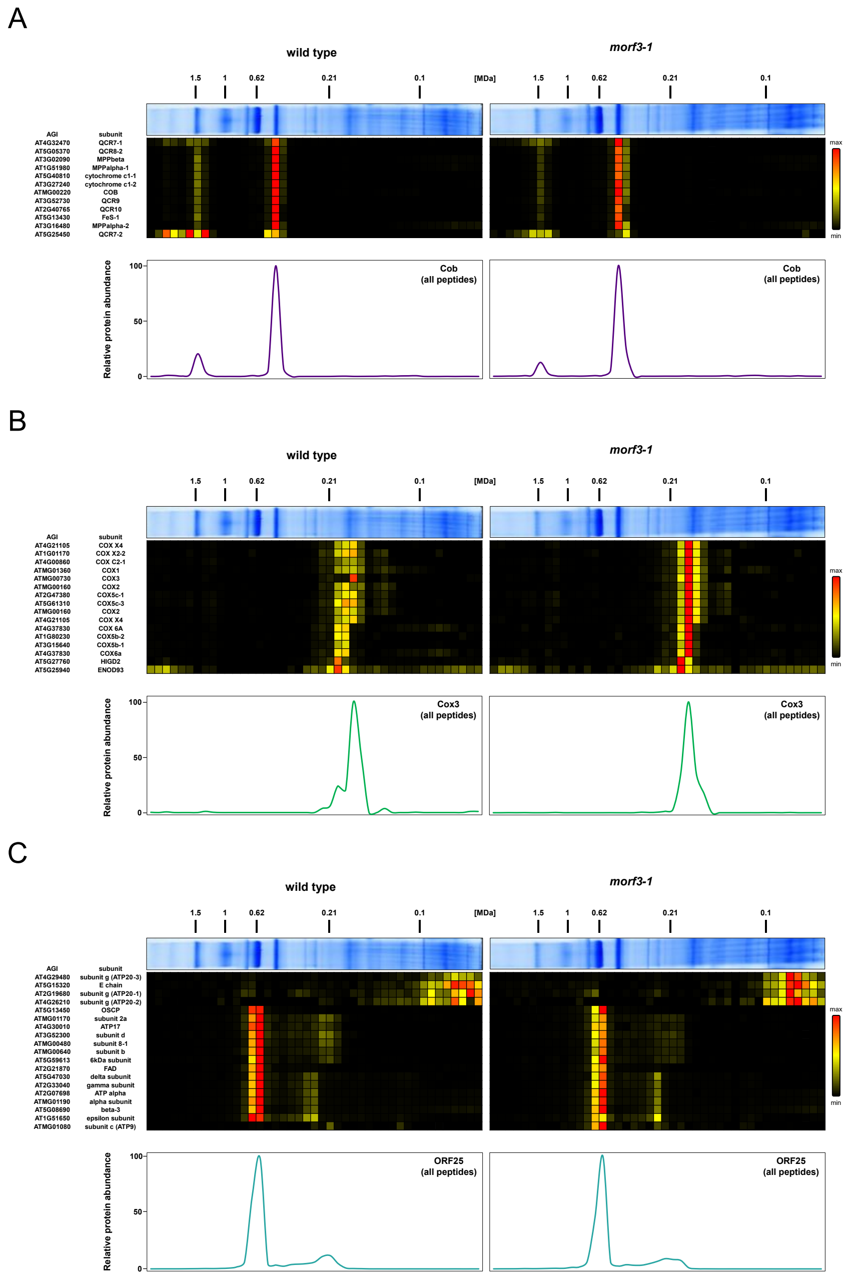

Supplementary Figure S7: **Abundance profiles of subunits of the complexes III, IV and V along BN-gel lanes in wild-type and *morf3-1* mutant Arabidopsis lines.** Mitochondrial protein fractions from *A. thaliana* wildtype and *morf3-1* mutant liquid cell cultures were separated on a Blue-Native (BN) gel. The gel lanes were segmented into 43 fractions and analyzed via mass spectrometry. The gel lanes on the top of each heatmap are identical to those shown in Figure 5A. For further information see legend of Figure 7. **A)** Abundance profiles of subunits of mitochondrial complex III. Upper panel: Heatmaps of proteins of mitochondrial complex III from wild type (left) and *morf3-1* mutant (right) lines. Each column represents a fraction of the BN gels. The rows describe the normalized abundance of individual proteins (based in iBAQ) along the gel lanes. Max abundance: red; 50% intensity: yellow; no detection: black. Lower panel: Normalized protein abundance profile of the cytochrome b (Cob) subunit based on summed-up intensities of all Cob peptides detected in the wild type and *morf3-1* mutant lines along the respective gel lanes. **B)** Abundance profiles of subunits of mitochondrial complex IV. Upper panel: Heatmaps of proteins of mitochondrial protein complex IV from wild type (left) and *morf3-1* mutant (right) lines. Each column represents a fraction of the BN gel. The rows describe the normalized abundance of individual proteins (based in iBAQ) along the gel lanes. Max abundance: red; 50% intensity: yellow; no detection: black. Lower panel: Normalized abundance profile of subunit III (Cox3) based on the summed-up intensities of all Cox3 peptides detected in the wild type and *morf3-1* mutant lines along the respective gel lanes. **C)** Abundance profiles of subunits of mitochondrial complex V. Upper panel: Heatmaps of proteins of mitochondrial protein complex V from wild type (left) and *morf3-1* mutant (right) lines. Each column represents a fraction of the BN gels. The rows describe the normalized abundance of individual proteins (based in iBAQ) along the gek lanes. Max abundance: red; 50% intensity: yellow; no detection: black. Lower panel: Normalized abundance profile of the ORF25 subunit of complex V based on the summed-up intensities of all ORF25 peptides detected in the wild type and *morf3-1* mutant lines along the respective gel lanes. The relative abundances of the three proteins in the lower panels was normalized to their respective maximum values (“100”). An accessible version of all complexome heatmaps for individuals with color vision deficiency can be found in Supplementary Table S4.

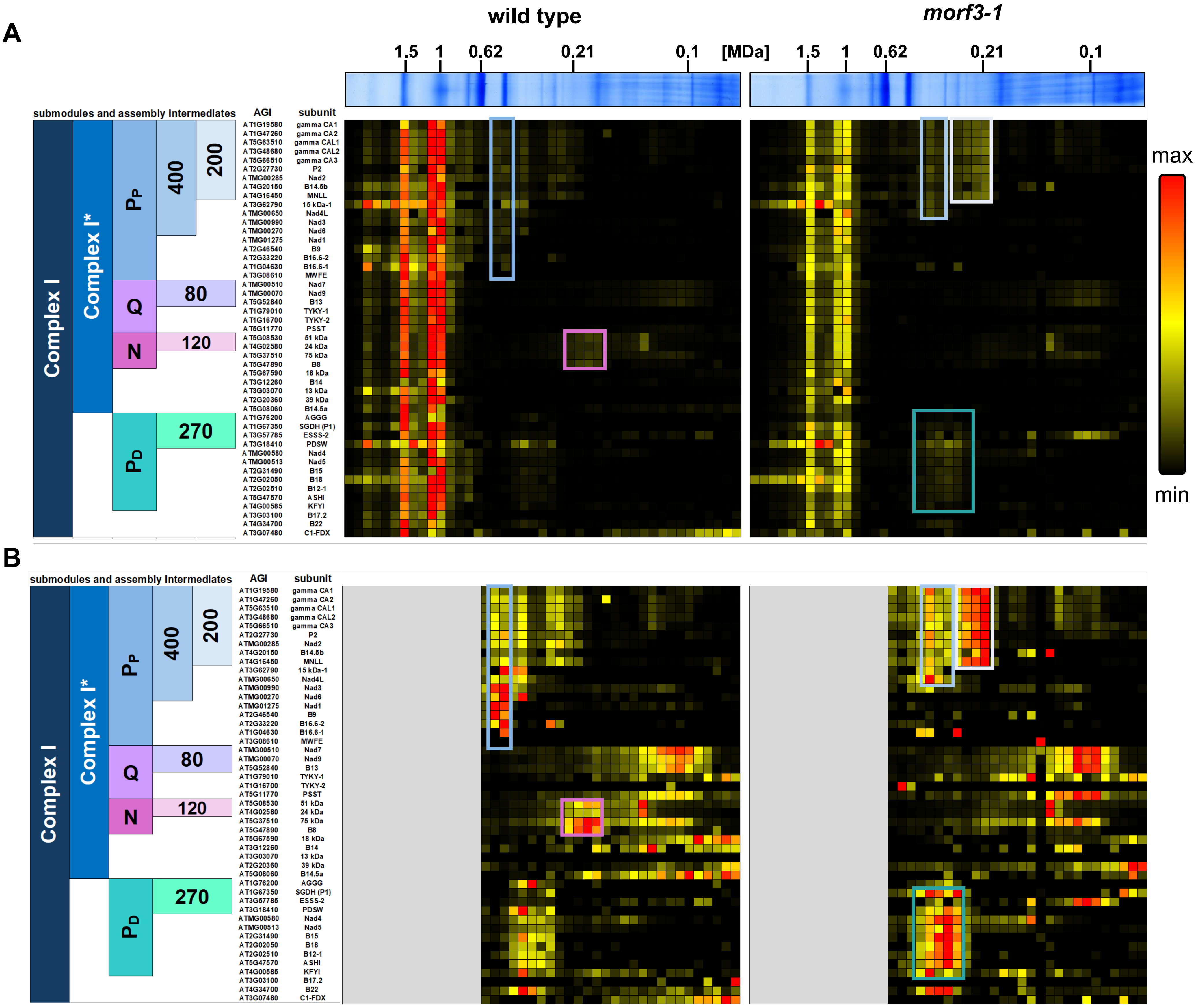

Supplementary Figure S8: Complex I assembly intermediates in wild-type and *morf3-1* mutant Arabidopsis plants as displayed by complexome profiling. A) The gel lanes on the top of the heatmap are identical to those shown in Figure 5A. Heatmap as shown in Figure 8: Abundance profiles of complex I subunits along a Blue-Native gel stripe of a mitochondrial sample from an Arabidopsis wild type line (left heatmap); abundance profiles of complex I subunits along a Blue-Native gel stripe of a mitochondrial sample from *morf3-1* mutant line (right heatmap); The columns of the heatmaps represent the fractions of the BN gel lanes. The rows display the normalized abundance profiles of the individual complex I subunits along the BN gel lanes. Max abundance: red; 50% intensity: yellow; no detection: black. Complex I subunits are sorted according their presence in complex I assembly intermediates (Ligas et al., 2019). Colored blocks to the left of the heatmaps illustrate assembly intermediates (submodules) of complex I. Assembly intermediates of increased abundance in one or the other mitochondrial sample are indicated by colored frames within the heatmaps (the color of the frames corresponds to those in the assembly intermediate (submodule) illustration to the very left). **B)** Same as part A) of the figure, but protein abundance profiles were recalculated for the 0-600 kDa range (fractions > 600 kDa were removed before the calculation). An accessible version of all complexome heatmaps for individuals with color vision deficiency can be found in Supplementary Table S4.

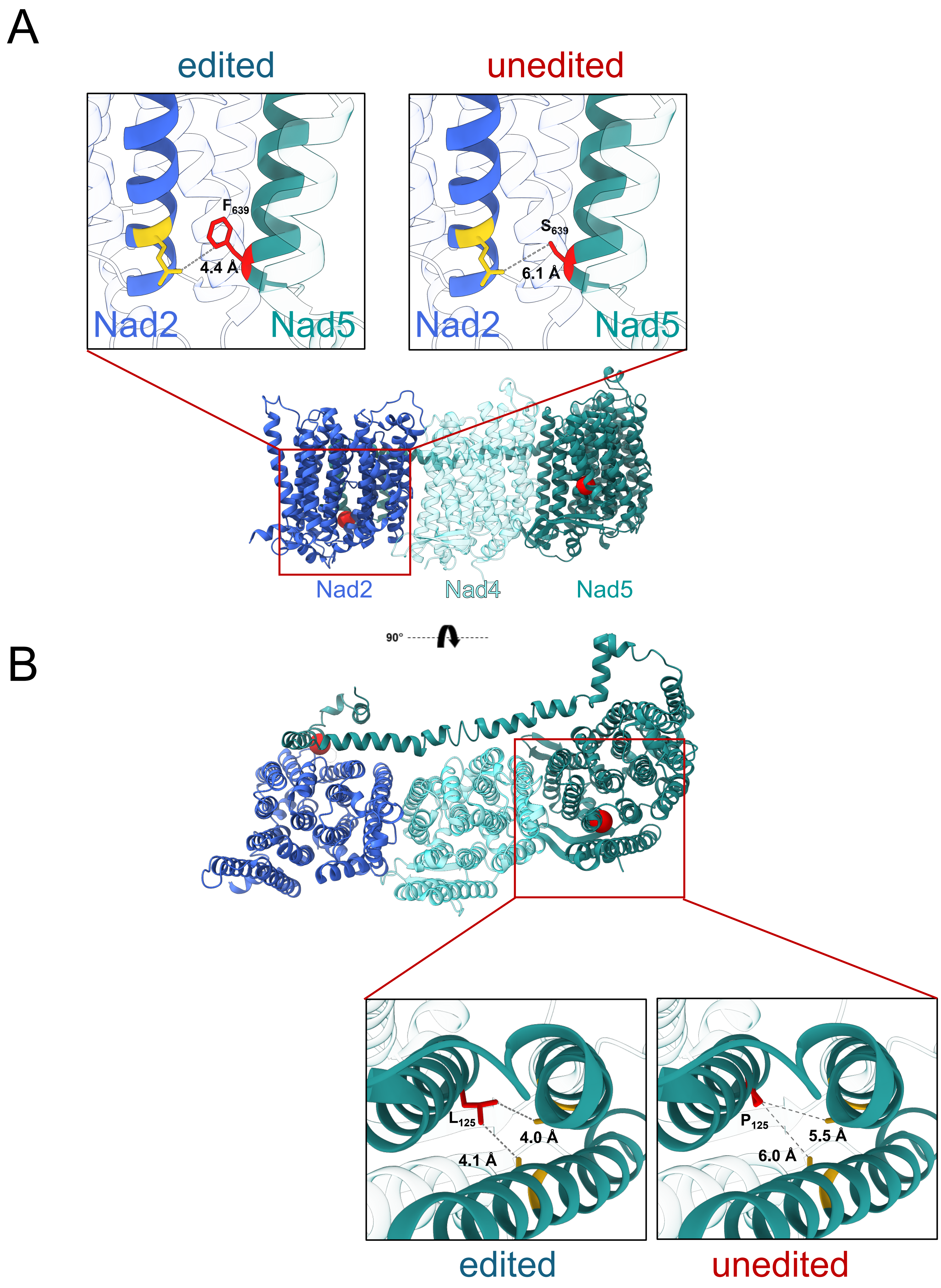

Supplementary Figure S9: Effects of disturbed Nad5 transcript editing on the structure of complex I in the *morf3-1* mutant line. A) Editing at the site nad5eU1916SFp100 causes a serine (upper right panel) to be replaced by a phenylalanine (upper left panel) at amino acid position 639 in the Nad5 subunit (turquoise). This amino acid position is located at the interphase of Nad2 (blue; Maldonado et al. 2022). RNA Editing frequency at this site is reduced by 50% in the *morf3-1* mutant line (Takenaka et al., 2012). **B)** Editing at the site nad5eU374PLp100 causes a proline (lower right panel) to be replaced by a leucine (lower left panel) at amino acid position 125 in the Nad5 subunit (turquoise). The remaining RNA editing frequency at this site is reported to be 60% in the *morf3-1* mutant line (Takenaka et al., 2012). This figure displays the protein structures based on the data from Klusch et al., 2023, which are available in the Protein Data Bank (PDB) under the ID 8BPX.

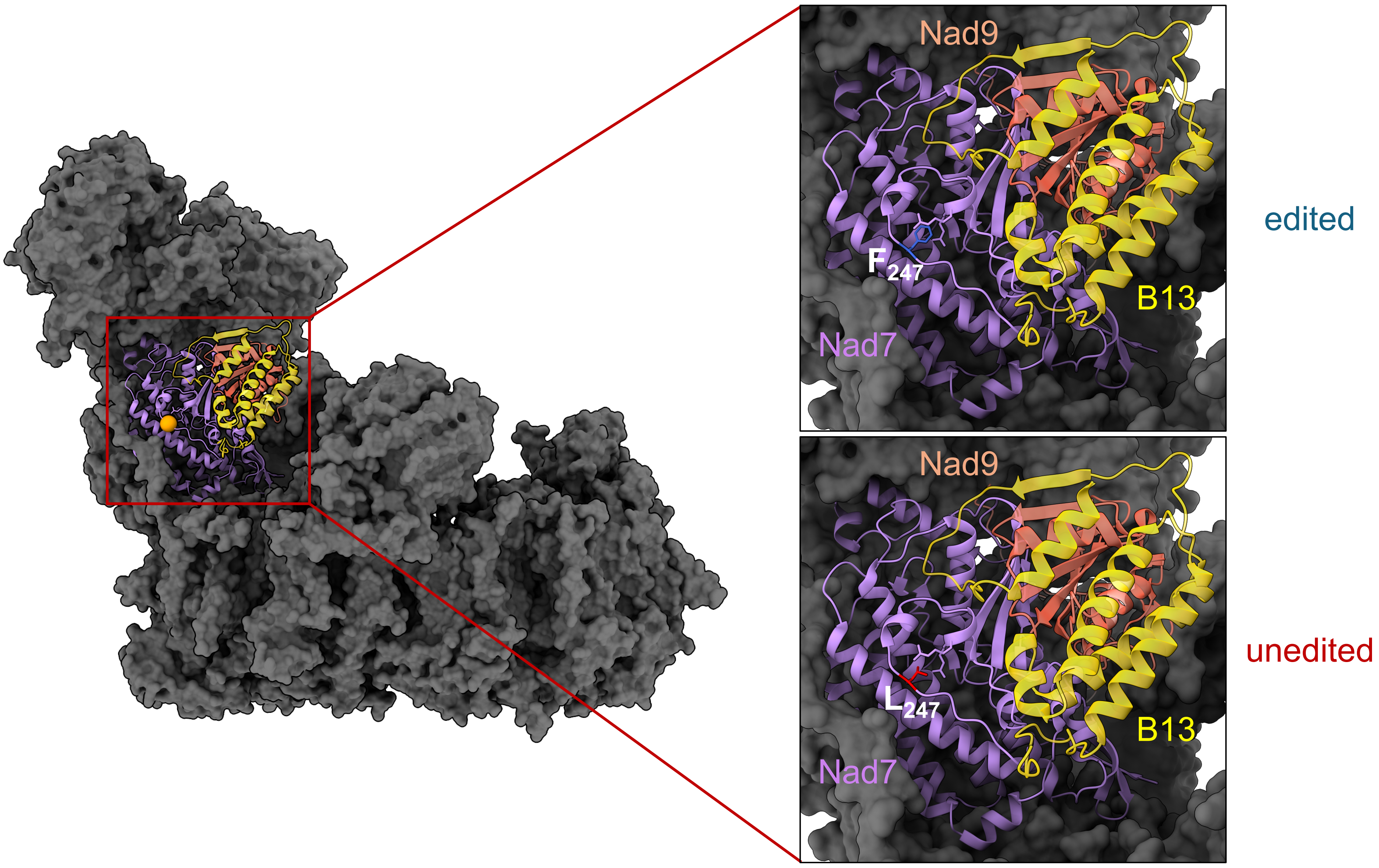

Supplementary Figure S10: Effects of disturbed Nad7 transcript editing on the structure of complex I in the *morf3-1* mutant line. Editing at the site nad7eU739LFp100 causes a leucine (red, lower right panel) to be replaced by a phenylalanine (blue, upper right panel) at amino acid position 247 in the Nad7 subunit (purple). This amino acid position has been reported to be located near a critical functional region within the complex (Maldonado et al., 2022). RNA Editing frequency at this site is reduced by 100% in the *morf3-1* mutant line (Takenaka et al., 2012; Supplementary Table S3). Together with Nad9 (orange) and B13 (yellow), Nad7 forms the 80 kDa precursor of the Q-module (120 kDa). The molecular surfaces of all other complex I subunits are shown in grey. This figure displays the protein structures based on the data from Klusch et al., 2023, which are available in the Protein Data Bank (PDB) under the ID 8BPX.

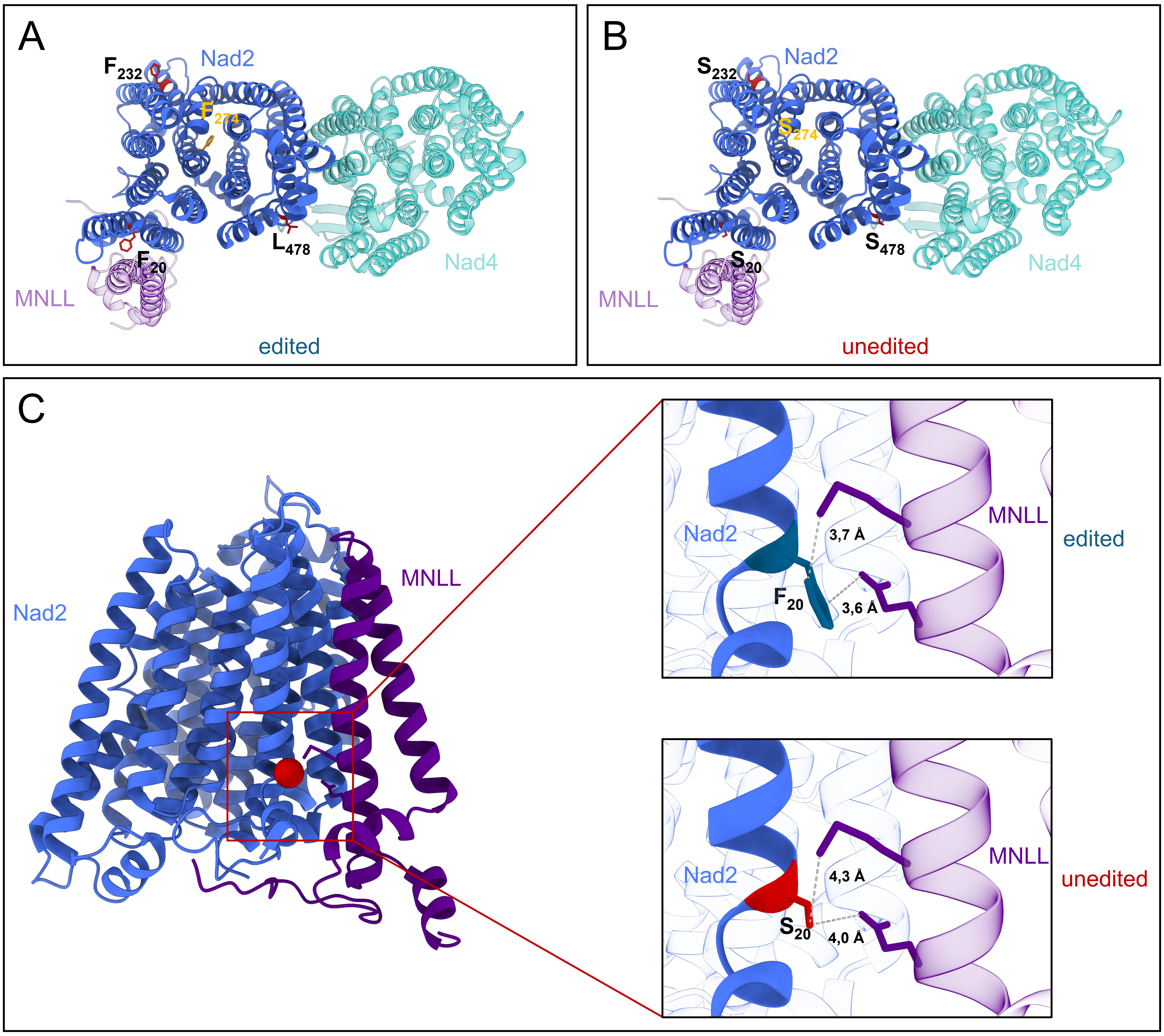

Supplementary Figure S11: Effects of disturbed Nad2 transcript editing on the structure of complex I in the *morf3-1* mutant line. Editing at four sites causes the replacement of serines (part **B** of the figure) by phenylalanines (part **A**) at the amino acid positions 20, 232, 274 and 478 in the Nad2 subunit (blue). The site characterized by our proteomics approach at amino acid position 274 is shown in yellow and is located centrally in the Nad2 subunit. RNA editing frequency was reported to be 0% in the *morf3-1* mutant (Takenaka et al. 2012; Supplementary Table S3). **C**) Amino acid position 20 is located at the interphase of the MNLL subunit (Maldonado et al. 2022). The remaining editing frequency of the respective RNA editing site nad2eU59SFp100 is 20% in the *morf3-1* mutant. Distances between the side chains at this interface are given in angstroms. For further details on all four Nad2 RNA editing sites, see Supplementary Table S3. This figure displays the protein structures based on the data from Klusch et al., 2023, which are available in the Protein Data Bank (PDB) under the ID 8BPX.

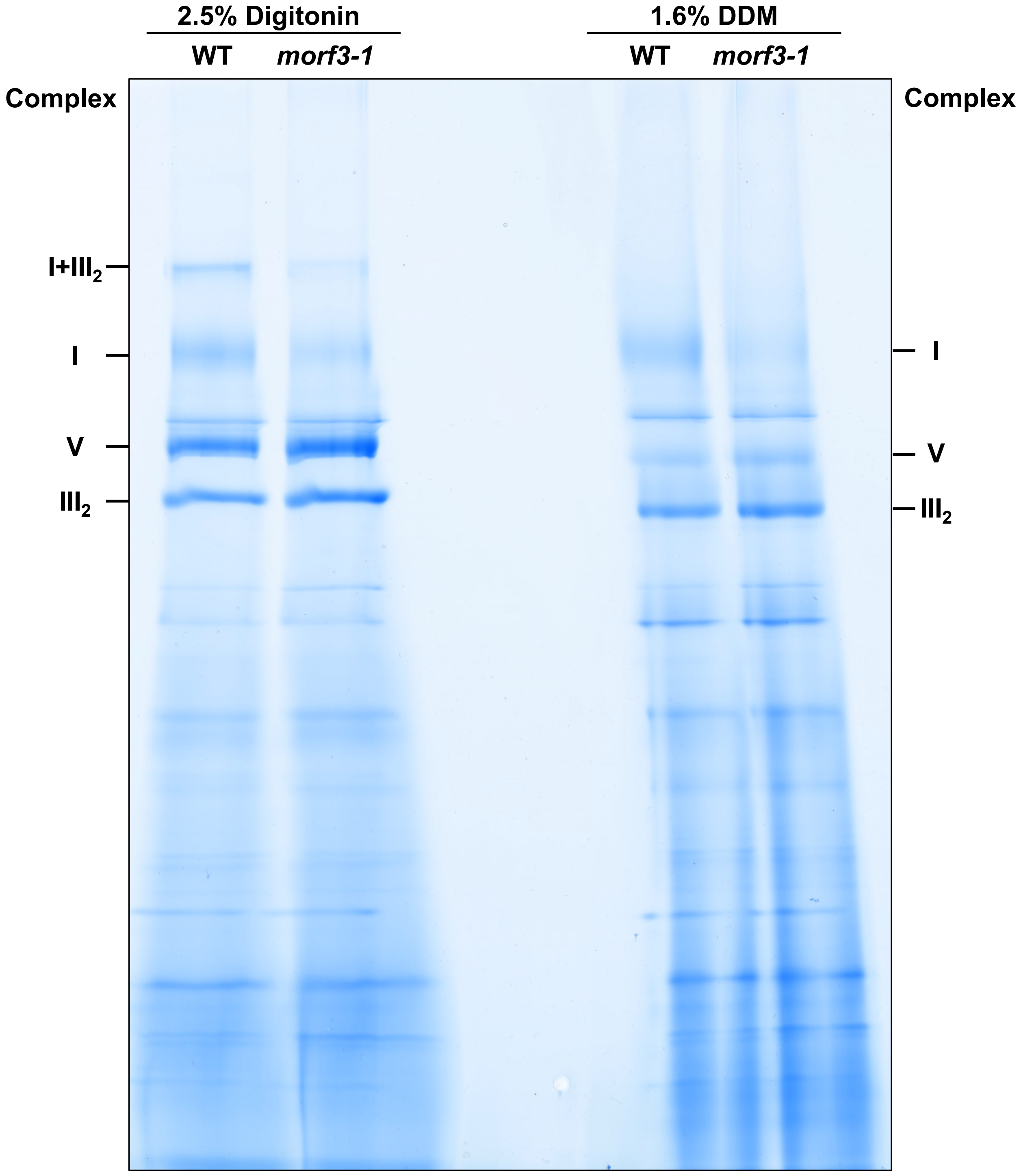

Supplementary Figure S12: Impact of solubilization conditions on the visibility of OXPHOS complexes from wild-type and *morf3-1* mutant lines upon their separation by 1D Blue-Native PAGE. Isolated mitochondria were solubilized using either 2.5% digitonin or 1.6% dodecyl maltoside (DDM). Proteins and protein complexes were separated by BN-PAGE as described in the “Materials and methods“ section. After completion of the gel electrophoresis run, the gel was stained with Coomassie brilliant blue. Identities of OXPHOS complexes are displayed to the left and right of the gel.

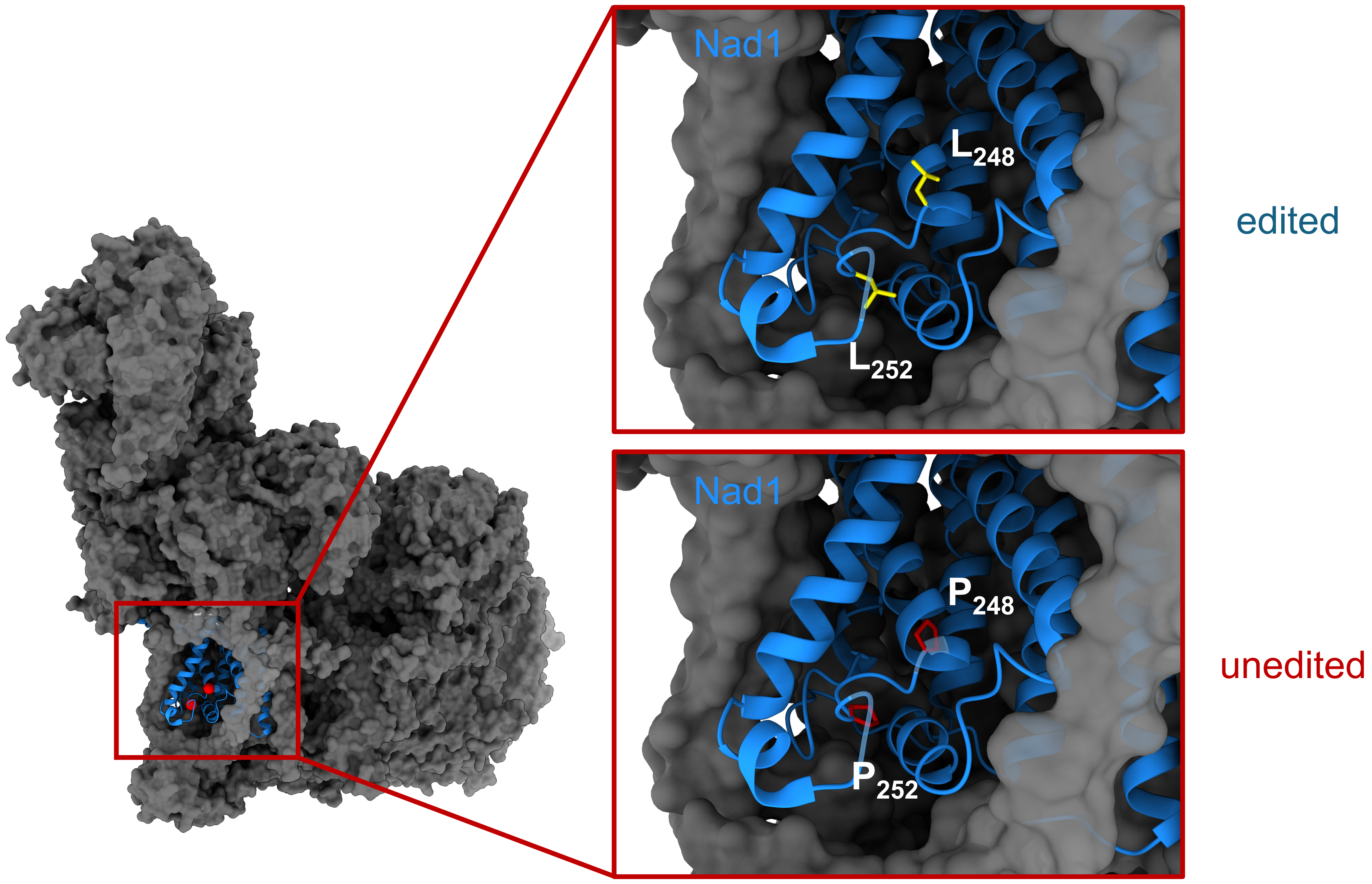

Supplementary Figure S13: Effects of disturbed Nad1 transcript editing on the structure of complex I in the *morf3-1* mutant line. Editing at sites nad1eU743PLp100 and nad1eU755PLp100 causes two adjacent proline residues (red, lower right panel) to be replaced by leucine residues at amino acid positions 248 and 252 (blue, upper right panel) in the Nad5 subunit (blue). The reported remaining RNA editing frequencies in the *morf3-1* mutant are 0% at nad1eU743PLp100 and 70% at nad1eU755PLp100 (Takenaka et al., 2012; Supplementary Table S3). Both sites were not reported to be located in an interface between Nad5 and other complex I subunits. The molecular surfaces of all other complex I subunits are shown in grey. This figure displays the protein structures based on the data from Klusch et al., 2023, which are available in the Protein Data Bank (PDB) under the ID 8BPX.
